# Artificial intelligence-enhanced electrocardiography derived body mass index as a predictor of future cardiometabolic disease

**DOI:** 10.1038/s41746-024-01170-0

**Published:** 2024-06-25

**Authors:** Libor Pastika, Arunashis Sau, Konstantinos Patlatzoglou, Ewa Sieliwonczyk, Antônio H. Ribeiro, Kathryn A. McGurk, Sadia Khan, Danilo Mandic, William R. Scott, James S. Ware, Nicholas S. Peters, Antonio Luiz P. Ribeiro, Daniel B. Kramer, Jonathan W. Waks, Fu Siong Ng

**Affiliations:** 1 National Heart and Lung Institute, Imperial College London, London, United Kingdom; 2 Department of Cardiology, Imperial College Healthcare NHS Trust, London, United Kingdom; 3 MRC Laboratory of Medical Sciences, Imperial College London, London, United Kingdom; 4 Department of Information Technology, Uppsala University, Uppsala, Sweden; 5 Chelsea and Westminster NHS Foundation Trust, London, United Kingdom; 6 Department of Electrical and Electronic Engineering, Imperial College London, London, United Kingdom; 7 Institute of Clinical Sciences, Faculty of Medicine, Imperial College London, London, United Kingdom; 8 Department of Internal Medicine, Faculdade de Medicina, and Telehealth Center and Cardiology Service, Hospital das Clínicas, Universidade Federal de Minas Gerais, Belo Horizonte, Brazil; 9 Richard A. and Susan F. Smith Center for Outcomes Research in Cardiology, Beth Israel Deaconess Medical Center, Harvard Medical School, Boston, MA USA; 10 Harvard-Thorndike Electrophysiology Institute, Beth Israel Deaconess Medical Center, Harvard Medical School, Boston, MA USA

**Keywords:** Prognostic markers, Machine learning, Obesity, Dyslipidaemias

## Abstract

The electrocardiogram (ECG) can capture obesity-related cardiac changes. Artificial intelligence-enhanced ECG (AI-ECG) can identify subclinical disease. We trained an AI-ECG model to predict body mass index (BMI) from the ECG alone. Developed from 512,950 12-lead ECGs from the Beth Israel Deaconess Medical Center (BIDMC), a secondary care cohort, and validated on UK Biobank (UKB) (*n* = 42,386), the model achieved a Pearson correlation coefficient (r) of 0.65 and 0.62, and an R^2^ of 0.43 and 0.39 in the BIDMC cohort and UK Biobank, respectively for AI-ECG BMI vs. measured BMI. We found delta-BMI, the difference between measured BMI and AI-ECG-predicted BMI (AI-ECG-BMI), to be a biomarker of cardiometabolic health. The top tertile of delta-BMI showed increased risk of future cardiometabolic disease (BIDMC: HR 1.15, *p* < 0.001; UKB: HR 1.58, *p* < 0.001) and diabetes mellitus (BIDMC: HR 1.25, *p* < 0.001; UKB: HR 2.28, *p* < 0.001) after adjusting for covariates including measured BMI. Significant enhancements in model fit, reclassification and improvements in discriminatory power were observed with the inclusion of delta-BMI in both cohorts. Phenotypic profiling highlighted associations between delta-BMI and cardiometabolic diseases, anthropometric measures of truncal obesity, and pericardial fat mass. Metabolic and proteomic profiling associates delta-BMI positively with valine, lipids in small HDL, syntaxin-3, and carnosine dipeptidase 1, and inversely with glutamine, glycine, colipase, and adiponectin. A genome-wide association study revealed associations with regulators of cardiovascular/metabolic traits, including *SCN10A*, *SCN5A*, *EXOG* and *RXRG*. In summary, our AI-ECG-BMI model accurately predicts BMI and introduces delta-BMI as a non-invasive biomarker for cardiometabolic risk stratification.

## Introduction

Obesity is a rapidly growing public health challenge^1^ and is a major contributor to the increasing incidence of cardiometabolic disease^2^. Body Mass Index (BMI) is commonly used to define obesity and the associated disease risk. However, BMI has known limitations as a derived variable, as it is an insensitive measure of visceral adiposity^3^ and fails to capture body fat location and total fat mass^4^, all of which are important determinants of cardiometabolic disease^5^. Consequently, BMI does not accurately reflect the risk of these diseases^6^. There is, therefore, a clear need for an accurate, easy-to-access biomarker that can capture the risk of cardiometabolic disease related to obesity and perform well across the entire population. This biomarker would ideally be able to measure the downstream effects of obesity, even in subclinical disease, to allow primary prevention treatments to be prescribed.

In recent years, the application of deep learning in the field of ECG has gained significant traction, driven by its remarkable diagnostic and predictive capabilities in predicting future cardiac diseases^7–10^. Obesity is associated with remodelling of cardiac electrophysiology, and there are well-described changes in the ECG associated with obesity, such as reduced voltage in precordial leads, T wave flattening, and QT interval prolongation^11–13^. With the emergence of AI-enhanced ECG, the potential for deep learning to extract relevant ECG features becomes even more compelling. Recent studies have demonstrated that AI-ECG algorithms are adept at capturing information not only on cardiac but also on non-cardiac diseases, including metabolic disorders and liver disease^14,15^. Building upon these findings, we hypothesised that the ECG contains valuable information about obesity and cardiometabolic risk, and that it could be accessed through deep learning.

Inspired by the recent success of AI-ECG-predicted age, where the “delta age” (i.e. the difference between the AI-predicted and chronological age) is associated with increased mortality^16^, we propose “delta-BMI” (the difference between AI-ECG predicted BMI and measured BMI), as a novel biomarker for cardiometabolic risk capable of identifying individuals at risk of future cardiometabolic disease, including diabetes, hypertension, and lipid disorders. Furthermore, we identified candidate biological pathways for this biomarker by exploring its phenotypic, genetic, metabolomic, and proteomic associations. Finally, using a form of unsupervised deep learning, a variational autoencoder, we explored the ECG morphological changes associated with the AI-ECG BMI predictions.

## Results

### AI-ECG BMI derivation

The Beth Israel Deaconess Medical Center (BIDMC) cohort, a secondary care dataset comprised of routinely collected data from Boston, USA, was used as the derivation cohort. In total, 512,950 ECGs from 114,415 subjects had paired BMI data available. The BIDMC dataset was divided into training, validation, and holdout test sets using a split ratio of 60/10/30%. Full details are provided in the Methods. Cohort demographics are shown in Supplementary Table 1.

**Fig. 1:**
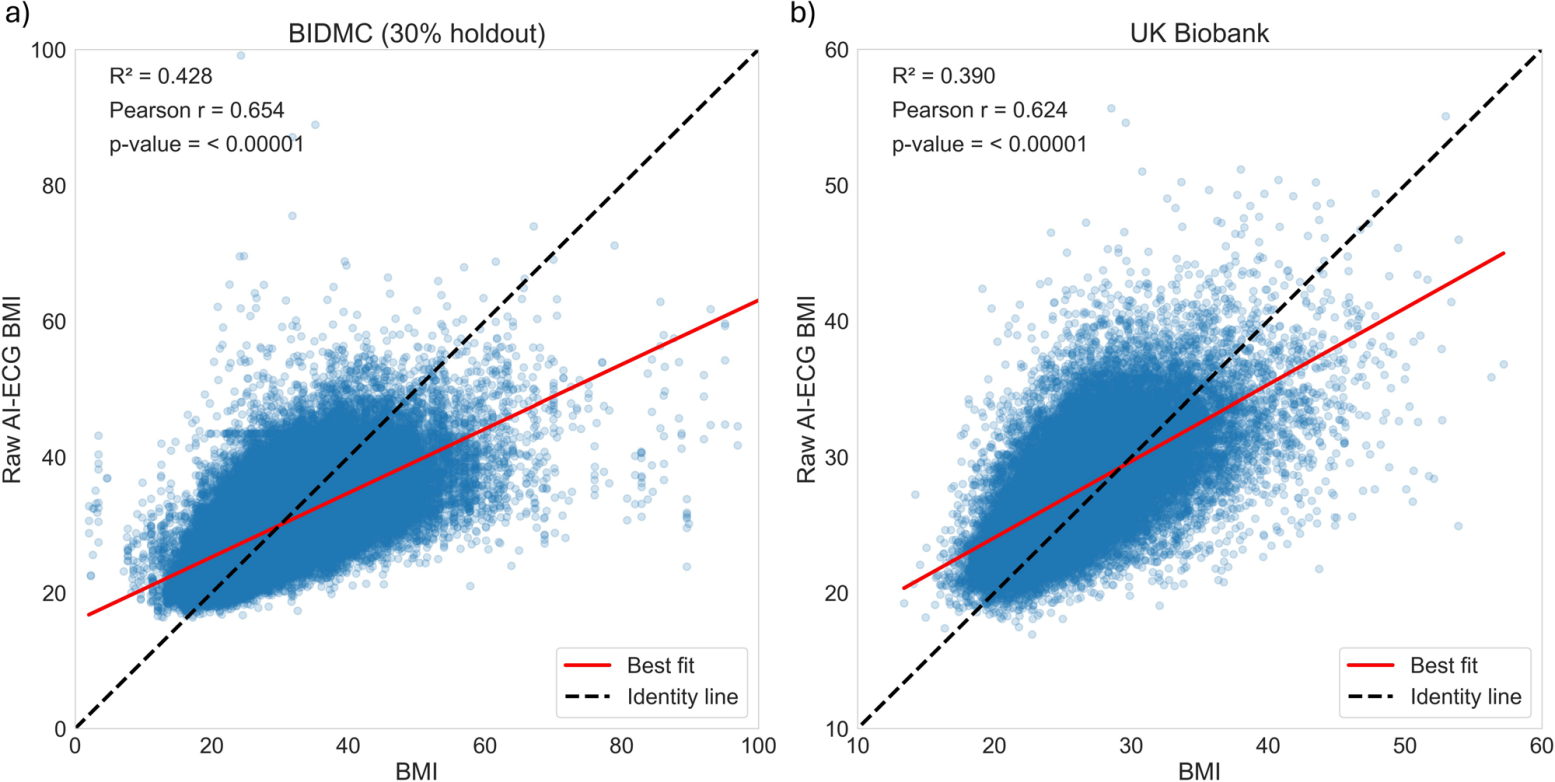
Association between AI-ECG BMI predictions and measured BMI in the BIDMC and UK Biobank cohorts. Scatter plots depicting the association between raw AI-ECG-BMI predictions and measured BMI within (**a**) the 30% holdout BIDMC and (**b**) UK Biobank cohorts. The black identity line serves as a reference point, representing the ideal prediction scenario. The red line represents the best-fit line. The R^2^ (Pearson correlation) was 0.43 (*r* = 0.65) in the 30% holdout BIDMC, and 0.39 (*r* = 0.62) in the UK Biobank cohort.

We trained the AI-ECG BMI model to predict the subject’s BMI on a continuous scale, using a previously described residual neural network (ResNet) architecture^17^. The AI-ECG-BMI model applied on the 30% holdout test set achieved a Pearson correlation coefficient of 0.65 (95% CI: 0.65–0.66) between measured BMI and AI-ECG predicted BMI, a correlation of determination (R^2^) of 0.43 (95% CI: 0.42–0.43), and a mean absolute error (MAE) of 3.95 (95% CI: 3.93–3.97) (Fig. 1, Supplementary Table 2).

### External validation

We used the UK Biobank, a healthy volunteer cohort^18^, to externally validate our findings. Digital ECGs and BMI measurements were available in 42,386 subjects. Cohort demographics are shown in Supplementary Table 1. Notably, as a volunteer cohort, the incidence of future adverse cardiometabolic events, except for hypertension, was lower in the UK Biobank as compared to the BIDMC cohort. In the UK Biobank external validation dataset, the AI-ECG-BMI model achieved a Pearson correlation coefficient of 0.62 (0.62–0.63), R^2^ of 0.39 (95% CI: 0.38–0.40), and an MAE of 2.94 (95% CI: 2.91–2.96) (Fig. 1, Supplementary Table 2). The correlation plots and Bland-Altman analyses for both cohorts are presented in Supplementary Fig. 1.

Supplementary Table 2 presents a comprehensive overview of the AI-ECG BMI model’s performance within the BIDMC and UK Biobank (UKB) cohorts, stratified across various subpopulations based on sex and ethnicity. Notably, the model demonstrates a better performance for females in both the BIDMC and UKB cohorts, as evidenced by the higher R^2^ values (BIDMC: *p* < 0.005, Δ *r* = 0.056, UKB: *p* < 0.005, Δ *r* = 0.041), i.e. the model’s predictions align more closely with actual BMI values for female participants. In terms of ethnicity, the model yields more accurate BMI predictions for African-American individuals in the BIDMC cohort compared to their Caucasian counterparts (*p* < 0.005, Δ *r* = 0.031), and less accurate predictions for Asian individuals compared to Caucasians (*p* < 0.005, Δ *r* = −0.065).

### Delta-BMI as a predictor of cardiometabolic disease

We calculated delta-BMI (AI-ECG predicted BMI minus measured BMI) to evaluate if this metric could provide additive prognostic information for future cardiometabolic disease, a composite of type 2 diabetes mellitus, hypertension, and lipid disorders (see outcome definitions in Supplementary Table 3). We performed Cox regression analyses with delta-BMI split into tertiles (bottom (delta-BMI ≤ −3.74), middle (−3.74 to 2.44), top (>2.44) (Table 1) and as a continuous variable (Supplementary Table 4), adjusting for measured BMI, age, and sex; therefore, our findings on delta-BMI can be considered additive to measured BMI.

**Table 1:** Adjusted hazard ratios of delta-BMI tertiles for future cardiometabolic outcomes

Type	High delta-BMI	95% CI	*p*-value	Middle delta-BMI	95% CI	*p*-value	Age	Female	BMI
BIDMC (holdout)									
Cardiometabolic disease	1.15	1.08–1.23	<0.001	1.12	1.06 – 1.20	<0.001	1.02	0.83	1.02
Type 2 Diabetes Mellitus	1.25	1.18–1.31	<0.001	1.10	1.05–1.16	<0.001	1.01	0.71	1.06
Hypertension	1.26	1.19–1.33	<0.001	1.17	1.11–1.24	<0.001	1.02	0.77	1.03
Lipid Disorders	1.21	1.15–1.27	<0.001	1.15	1.10–1.21	<0.001	1.02	0.78	1.03

BIDMC (holdout) – Cardiometabolic Disease									
Outpatient, BMI 18.5–24.9	1.31	1.10–1.57	0.003	1.11	0.95–1.29	0.194	1.03	0.82	1.04
Outpatient, BMI > 25	1.20	1.07–1.36	0.003	1.27	1.13–1.43	<0.001	1.01	0.97	1.01
Outpatient, BMI > 30	1.28	1.07–1.53	0.008	1.23	1.02–1.48	0.029	1.00	1.00	1.01
Outpatient, Females	1.37	1.21–1.56	<0.001	1.19	1.06–1.34	0.005	1.02	N/A	1.02
Outpatient, Males	1.12	0.96–1.30	0.158	1.28	1.11–1.47	<0.001	1.01	N/A	1.01

UK Biobank									
Cardiometabolic disease	1.58	1.41–1.76	<0.001	1.36	1.22–1.51	<0.001	1.06	0.72	1.09
Type 2 Diabetes Mellitus	2.28	1.76–2.96	<0.001	1.53	1.17–2.00	0.002	1.04	0.51	1.16
Hypertension	1.54	1.37–1.75	<0.001	1.33	1.18–1.50	<0.001	1.06	0.72	1.10
Lipid Disorders	1.56	1.32–1.85	<0.001	1.46	1.24–1.71	<0.001	1.06	0.61	1.06

UK Biobank – Cardiometabolic Disease									
BMI 18.5–24.9	1.63	1.31–2.03	<0.001	1.57	1.29–1.90	<0.001	1.08	0.81	1.12
BMI > 25	1.52	1.33–1.73	<0.001	1.26	1.10–1.43	<0.001	1.05	0.69	1.08
BMI > 30	1.56	1.27–1.91	<0.001	1.28	1.05–1.57	0.017	1.05	0.68	1.07
Females	1.73	1.47–2.03	<0.001	1.45	1.24–1.69	<0.001	1.07	N/A	1.09
Males	1.44	1.24–1.68	<0.001	1.26	1.09–1.46	0.002	1.06	N/A	1.09

Hazard Ratios from survival analysis of delta-BMI tertiles adjusted for measured BMI, age, and sex, on the future incidence of cardiometabolic disease, type 2 diabetes mellitus, hypertension, and lipid disorders in the BIDMC holdout set and the UK Biobank cohort. Sub-analyses include cardiometabolic disease stratified by BMI categories (18.5–24.9, ≥ 25, ≥30) and sex for both the BIDMC holdout set and the UK Biobank cohort. Tertile cut-offs for delta-BMI were defined as follows: Bottom (delta-BMI ≤ 3.74), Middle (−3.74 to 2.44), and Top (>2.44). The analysis excluded BMI < 18.5 due to insufficient numbers of participants.

**Fig. 2:**
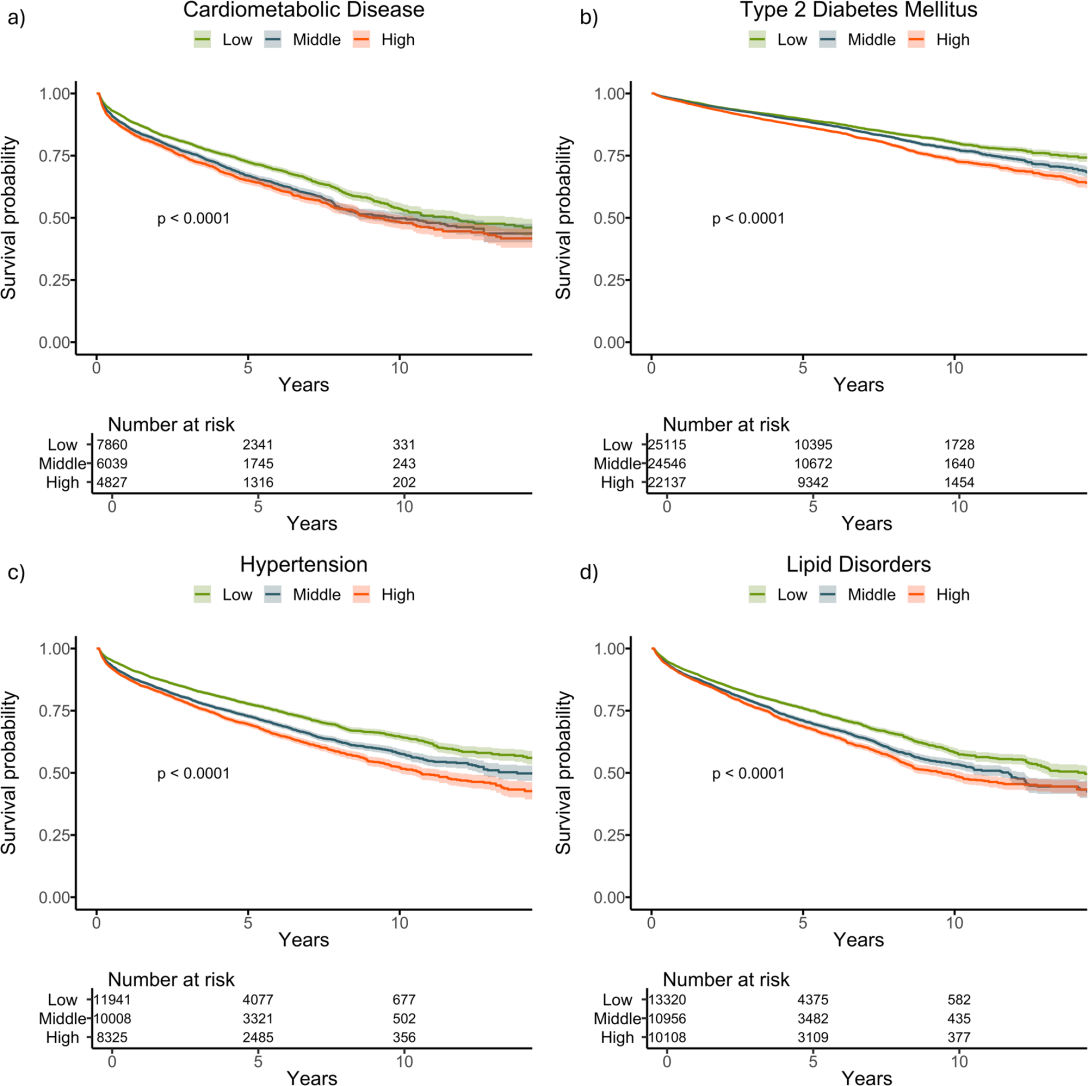
Kaplan–Meier survival curves stratified by delta-BMI curves for future cardiometabolic outcomes in the BIDMC cohort. Kaplan–Meier survival curves stratified by tertiles of delta-BMI in the BIDMC Cohort: Subplots **a**–**d** depict survival curves for cardiometabolic disease, type 2 diabetes mellitus, hypertension, and lipid disorders, respectively. Patients are stratified into tertiles based on delta-BMI, providing insights into the differential risk of each outcome. Log-rank *p*-values are reported for each outcome, highlighting statistically significant differences in survival across delta-BMI tertiles. Tertile cut-offs for delta-BMI are defined as follows: Bottom *(*delta-BMI ≤ −3.74), Middle (−3.74 to 2.44), and Top (>2.44).

In the BIDMC holdout dataset, participants in the top tertile of delta-BMI exhibited a 15% higher risk for the development of future cardiometabolic disease compared to those in the bottom tertile (Hazard ratio (HR) 1.15, 95% CI: 1.08–1.23, *p* < 0.001) (Table 1). These patterns were consistent for individual components of cardiometabolic disease, with higher risks observed for type 2 diabetes mellitus (T2DM) (HR: 1.25, 95% CI: 1.18–1.31, *p* < 0.001), hypertension (HR: 1.26, 95% CI: 1.19–1.33, *p* < 0.001), and lipid disorders (HR: 1.21, 95% CI: 1.15–1.27, *p* < 0.001), in the top tertile of delta-BMI, compared with the bottom tertile. Survival curves for the BIDMC cohort are depicted in Fig. 2. Importantly, these associations were identified regardless of whether the measured BMI was in the healthy weight, overweight, or obese ranges (Table 1).

**Fig. 3:**
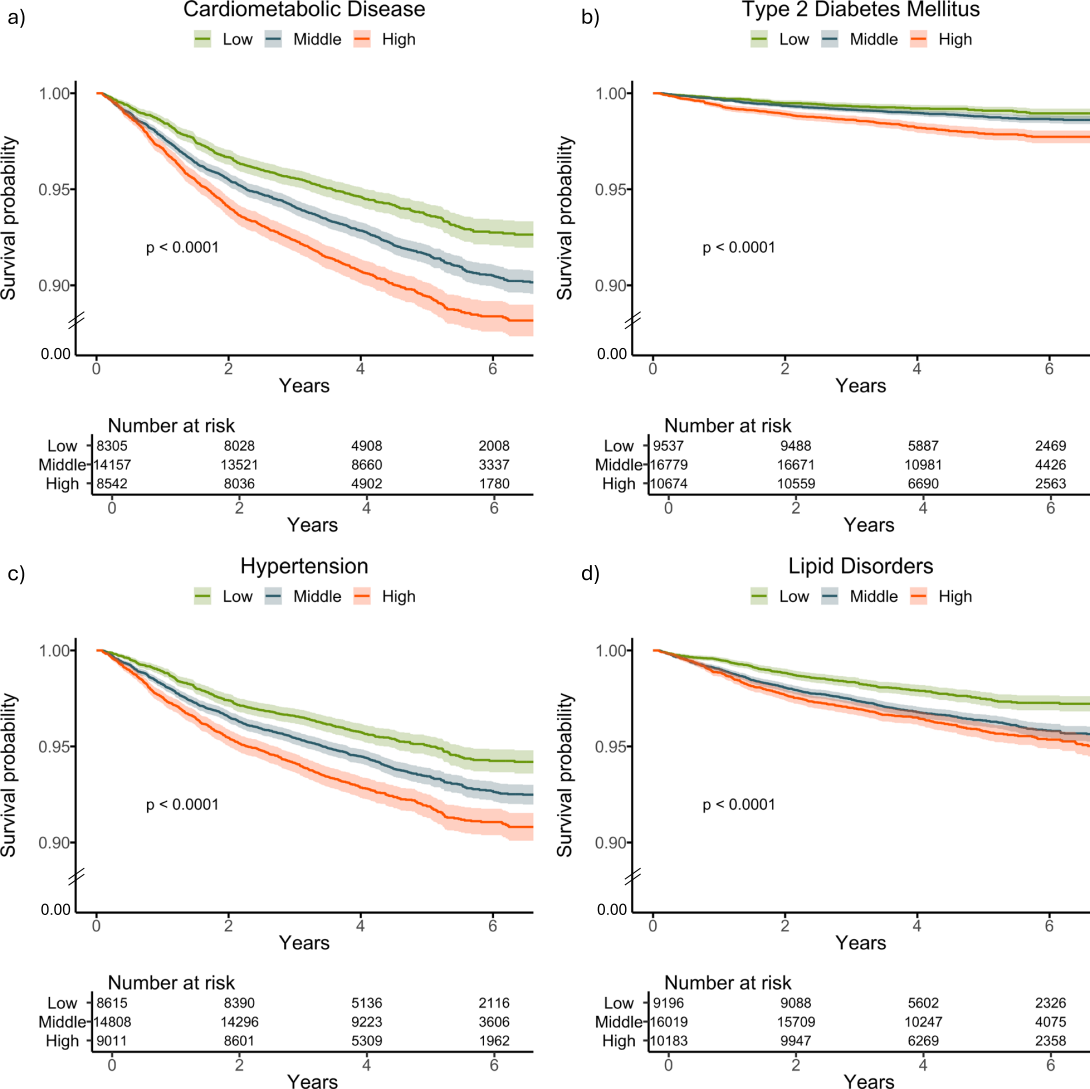
Kaplan–Meier survival curves stratified by delta-BMI curves for future cardiometabolic outcomes in the UK Biobank. Kaplan–Meier survival curves stratified by tertiles of delta-BMI in the UK Biobank Cohort**:** Subplots **a**–**d** depict survival curves for cardiometabolic disease, type 2 diabetes mellitus, hypertension, and lipid disorders, respectively. Patients are stratified into tertiles based on delta-BMI, providing insights into the differential risk of each outcome. Log-rank *p*-values are reported for each outcome, highlighting statistically significant differences in survival across delta-BMI tertiles. Tertile cut-offs for delta-BMI are defined as follows: Bottom *(*delta-BMI ≤ *−*3.74), Middle (−3.74 to 2.44), and Top (>2.44). To enhance clarity, the lower limit of the y-axis has been adjusted to 0.90, indicated by the break lines between 0.90 and 0.

In the UK Biobank cohort, individuals within the top tertile of delta-BMI demonstrated a 1.58-fold increased risk (95% CI: 1.41–1.76, *p* < 0.001) of developing future cardiometabolic diseases compared to those in the bottom tertile, after adjusting for measured BMI, age, and sex. Furthermore, participants in the top tertile of delta-BMI had a 2.28-fold increased risk (95% CI: 1.76-2.96, *p* < 0.001) of developing future T2DM, a 1.54-fold increased risk of developing future hypertension (HR 1.54, 95% CI: 1.37-1.75, *p* < 0.001) and a 1.56-fold risk of developing future lipid disorders (HR 1.56, 95% CI: 1.32-1.85, *p* < 0.001). The survival curves for the UK Biobank cohort are shown in Fig. 3. Notably, these associations were observed again irrespective of the BMI category or sex (Table 1).

We also found significant associations between delta-BMI, as a continuous variable, and incident cardiometabolic disease, type 2 diabetes mellitus, hypertension, and lipid disorders for both BIDMC and UKB cohorts (Supplementary Table 4).

Furthermore, we assessed the added predictive value of delta-BMI in cardiometabolic disease and T2DM prediction through likelihood ratio tests and continuous net reclassification analyses, while also examining changes in the concordance index upon its inclusion in the Cox model (Supplementary Table 5). Incorporating delta-BMI resulted in significant enhancements in model fit for cardiometabolic disease, T2DM, hypertension, and lipid disorders, as indicated by the likelihood ratio tests. Additionally, significant improvements in continuous net reclassification indices (NRI) were observed across both the BIDMC and UK Biobank cohorts. Minor yet significant enhancements in discriminatory power were evident in both cohorts. Notably, within the BIDMC cohort, subgroups with higher BMI (>25 and >30) exhibited notable improvements in the concordance index for future cardiometabolic disease (ΔC-index = 0.0098 (0.0020–0.0177) and ΔC-index = 0.0362 (0.0218–0.0755), respectively). Looking at the components of the cardiometabolic disease, the most notable improvement in model fit, assessed by the likelihood ratio test and the continuous NRI, was observed for T2DM in both cohorts. For BIDMC outpatients with a BMI of 18.5–24.9, significant enhancements in C-index were observed (ΔC-index = 0.0239 (0.0053–0.0377)), with similar findings in the UK Biobank cohort (ΔC-index = 0.0377 (0.0036–0.0751)).

Furthermore, we have explored the additive effect of delta-BMI in the different ethnic groups in the BIDMC cohort. Delta-BMI significantly improved the model fit and reclassification of cardiometabolic disease in Caucasians and Hispanics and of T2DM in Caucasians and Asians.

### Biological plausibility: clinical associations of delta-BMI

**Fig. 4:**
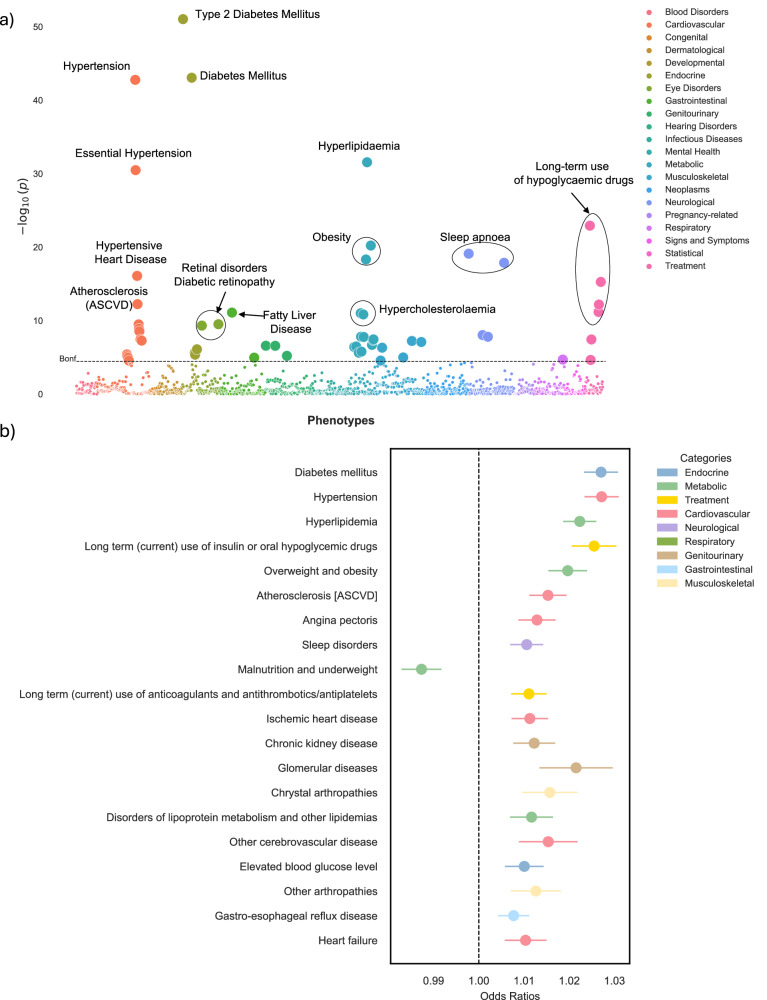
Phenome-wide association study (PheWAS) of delta-BMI in the BIDMC cohort. Exploration of the underlying biology through a phenome-wide association study (PheWAS) in the BIDMC cohort: **a** A PheWAS Manhattan plot showing the negative logarithm of the univariate logistic regression *p*-values between delta-BMI and disease phecodes, adjusted for measured BMI, sex, age, and age^2^. The dashed horizontal line signifies the Bonferroni corrected threshold for multiple comparisons. Out of 1408 comparisons, 55 (3.9%) reached significance based on the Bonferroni correction. An interactive version of the plots can be accessed in the Online Supplement. **b** Illustrates the top 20 significant phecodes associated with delta-BMI, presenting their respective odds ratios with 95% CI. ASCVD atherosclerotic cardiovascular disease.

To investigate the underlying associations between delta-BMI and different diseases, we performed a phenome-wide association (PheWAS) study in the BIDMC cohort. To do this, we performed univariate logistic regression analyses between delta-BMI (adjusted for measured BMI, sex, age, and age^2^), and 1408 different phecodes with at least 100 cases. Given the adjustment for measured BMI, our findings should be considered additive to traditional measured BMI. A Manhattan plot depicts 55 (3.9%) significant associations in Fig. 4a. An interactive version of the figure can be accessed in the Online Supplement. We identified significant associations with type 2 diabetes mellitus, hypertension, hypertensive heart disease, long-term (current) use of insulin and oral hypoglycaemics, hyperlipidaemia, hypercholesterolaemia, liver disease, and ASCVD. Figure 4b presents odds ratios (ORs) per unit change of delta-BMI for the top 20 significant top-level phecodes.

### Biological plausibility: phenotypic associations of delta-BMI

**Fig. 5:**
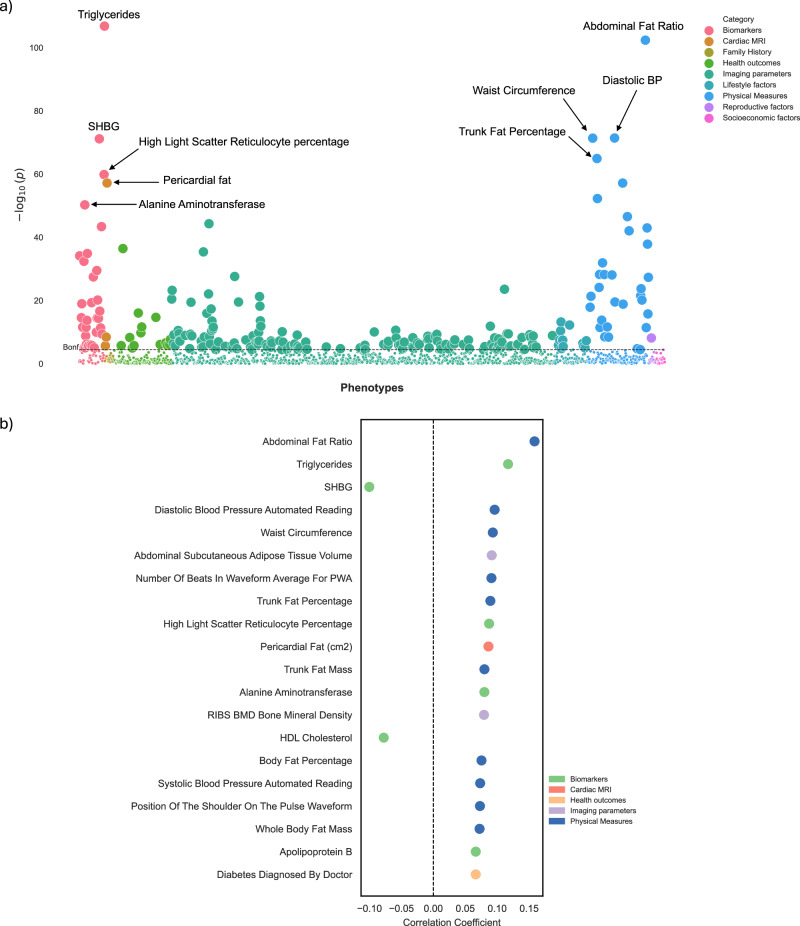
Phenome-wide association study (PheWAS) of delta-BMI in the UK Biobank. Exploration of the underlying biology through a phenome-wide association study (PheWAS) in the UK Biobank using clinical phenotypes: **a** A PheWAS Manhattan plot showing the negative logarithm of the univariate correlation *p*-values between delta-BMI and routinely recorded clinical features, adjusted for measured BMI, sex, age, and age^2^. The dashed horizontal line signifies the Bonferroni corrected threshold for multiple comparisons. Out of 1368 comparisons, 231 (16.9%) reached significance based on the Bonferroni correction, most of which came from imaging parameters, physical measures, and biomarkers. An interactive version of the plots can be accessed in the Online Supplement. **b** Illustrates the top 20 significant clinical phenotypes correlated with delta-BMI, presenting their respective correlation coefficients (Pearson). SHBG Sex Hormone Binding Globulin, PWA Pulse Wave Analysis, BP Blood Pressure, BMD Bone Mineral Density, HDL High-Density Lipoprotein.

Subsequently, we performed a UK Biobank phenome-wide association analysis between 1368 phenotypes and delta-BMI, adjusted for BMI, sex, age, and age^2^. The phenotype dataset included a wide array of categories, such as blood tests, cardiac and brain MRI features, imaging parameters, physical measures, and others. A Manhattan plot depicts all the significant associations in Fig. 5a (an interactive version can be accessed in the Online Supplement). After correcting for the Bonferroni threshold of significance, we identified 231 (16.9%) statistically significant phenotypes. These included key biomarkers, such as triglycerides, high light scatter reticulocyte percentage, alanine aminotransferase, and apolipoprotein B. For physical measures, positive associations included abdominal fat ratio, diastolic blood pressure, and waist circumference. Delta-BMI was also positively associated with imaging parameters, such as abdominal subcutaneous adipose tissue volume and bone mineral density. Furthermore, cardiac MRI associations included pericardial fat mass. We also identified significant negative associations between delta-BMI and sex hormone-binding globulin and HDL cholesterol (Fig. 5b).

### Biological plausibility: metabolomic associations of delta-BMI

**Fig. 6:**
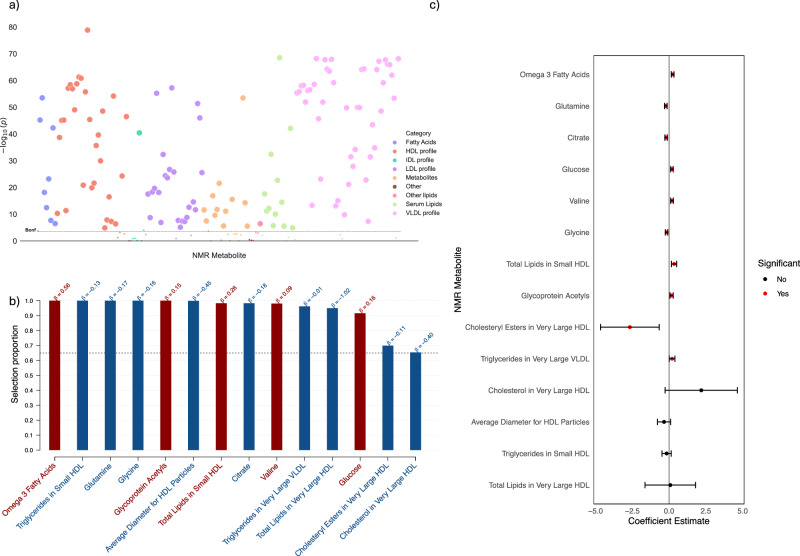
Metabolomic analysis of delta-BMI variability. Exploration of the underlying biology of delta-BMI variability using the UK Biobank NMR metabolomic data: **a** A metabolome-wide association study (MWAS) Manhattan plot showing the negative logarithm of the univariate correlation p-values between delta-BMI and the concentrations of NMR metabolites, adjusted for BMI, sex, age, and age^2^. Out of 168 comparisons, 136 (80.1%) reached significance based on the Bonferroni correction. An interactive version of the plots can be accessed in the Online Supplement. **b** Stability selection analysis employing LASSO regression on significant MWAS metabolites: This analysis, conducted over 1000 iterations with 80% subsampling, identifies robust metabolite associations with delta-BMI. Adjustments were made for measured BMI, sex, age, and age^2^. The black dashed line represents the calibrated selection proportion. **c** Multivariate linear regression analysis of stably selected metabolites against delta-BMI, adjusted for measured BMI, sex, age, and age^2^, demonstrating the individual contribution of stably selected metabolites to variations in delta-BMI.

In our metabolomic profiling analysis, we used a metabolome-wide association study (MWAS) as an initial step in selecting metabolites associated with delta-BMI (Fig. 6a). The significant variables (*n* = 136) were then used in a stability selection with LASSO (Fig. 6b). The resulting stably selected metabolites (*n* = 14) were used in a multivariate linear regression to identify their contributions to delta-BMI variability (Fig. 6c). We found significant negative associations between delta-BMI and glutamine, citrate, glycine, and cholesteryl esters in very large HDL. Significant positive associations were found between delta-BMI and omega-3 fatty acids, valine, glucose, glycoprotein acetyls, total lipids in small HDL, and triglycerides in very large VLDL. Collectively, these metabolites explained a modest proportion of delta-BMI variability (R^2^ = 0.046).

### Biological plausibility: proteomic associations of delta-BMI

**Fig. 7:**
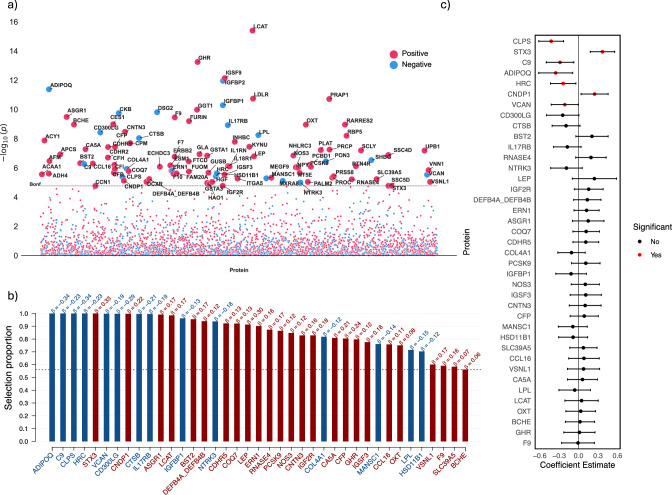
Proteomic analysis of delta-BMI variability. Exploration of the underlying biology of delta-BMI variability using the UK Biobank PPP data: **a** A protein-wide association study (PWAS) Manhattan plot showing the negative logarithm of the univariate correlation *p*-values between delta-BMI and the concentration of proteins, adjusted for measured BMI, sex, age, and age^2^. Of the 2919 proteins analysed, 100 (3.4%) surpassed the Bonferroni-corrected significance threshold. An interactive version of the plots can be accessed in the Online Supplement. **b** Stability selection analysis employing LASSO regression on significant PWAS proteins: This analysis, conducted over 1000 iterations with 80% subsampling, identifies robust protein associations with delta-BMI. Adjustments were made for measured BMI, sex, age, and age^2^. The black dashed line represents the calibrated selection proportion. **c** Multivariate linear regression analysis of stably selected proteins against delta-BMI, adjusted for measured BMI, sex, age, and age^2^, demonstrating the individual contribution of stably selected proteins to variations in delta-BMI.

In the proteomic analysis, we first conducted a proteome-wide association study (PWAS, Fig. 7a), identifying 100 significant proteins. Next, stability selection via LASSO (Fig. 7b) identified 39 stably selected proteins. Finally, we used multivariate linear regression with these proteins to evaluate their impact on delta-BMI variability (Fig. 7c). We found significant negative associations between delta-BMI and colipase (CLPS), complement C9 (C9), adiponectin (ADIPOQ), histidine-rich calcium-binding proteins (HRC), and versican (VCAN). Significant positive associations were found between delta-BMI and syntaxin 3 (STX3) and carnosine dipeptidase 1 (CNDP1). Collectively, these proteins explained a higher proportion of delta-BMI variability (R^2^ = 0.098) than the NMR metabolites.

### Biological plausibility: genetic associations of delta-BMI

**Fig. 8:**
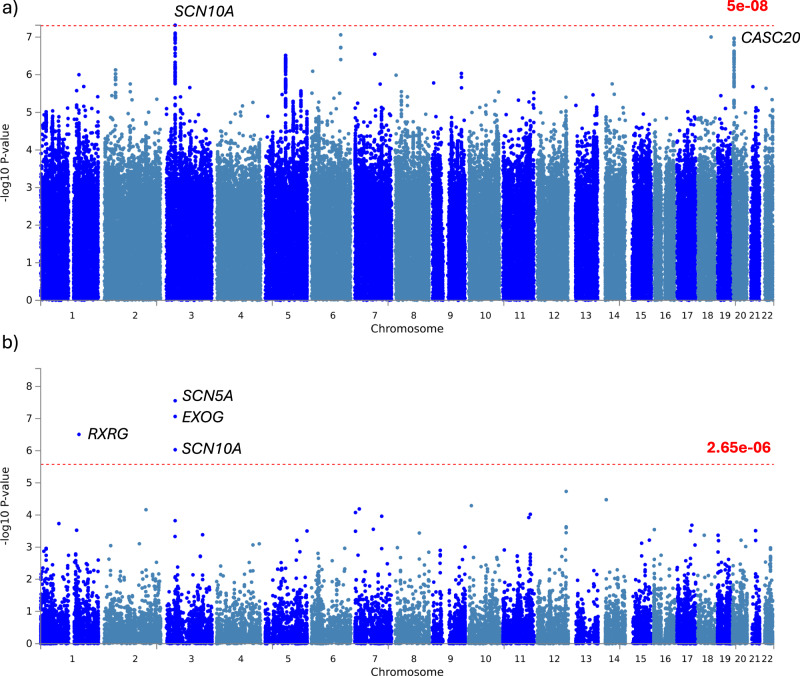
Genome-wide association study (GWAS) of delta-BMI variability. Exploration of the underlying biology of delta-BMI variability through a genome-wide association study (GWAS): GWAS Manhattan plots of genomic loci associated with delta-BMI. **a** Highlights the nearest genes associated with single nucleotide polymorphisms (SNP), with the red line depicting the genome-wide significant threshold (*P* < 5 ×10^−8^). **b** Displays a Manhattan plot derived from the gene-based test using MAGMA, mapping input SNPs to 18,882 protein-coding genes; the red line represents the genome-wide significant threshold (*P* < 2.65 ×10^−6^). SCN10A sodium voltage-gated channel alpha subunit 10, CASC20 cancer susceptibility 20, RXRG retinoid X receptor gamma, SCN5A sodium voltage-gated channel alpha subunit 10, EXOG exo/endonuclease G.

To investigate the underlying genetic associations with delta-BMI, we performed a genome-wide association study (Fig. 8a). We found a significant locus adjacent to sodium voltage-gated channel alpha subunit 10 (*SCN10A*), and a borderline significant locus adjacent to cancer susceptibility 20 (*CASC20*). Additionally, we used the summary GWAS characteristics to test for gene-level associations and identified additional associations with retinoid X receptor gamma *(RXRG)* and exo/endonuclease G *(EXOG)* (Fig. 8b). *SCN10A* is a regulator of prolonged PR interval^19^, Brugada syndrome^20^, and ECG morphology^21–23^. The top 3 variants of *SCN10A* in our analysis have previously been associated at GWAS with resting heart rate^24^, heart rate response to exercise^25^, and P wave duration^26^. *CASC20* is a regulator of body height, BMI-adjusted waist circumference, and heel-bone mineral density (a measure of osteoporosis). The lead variant in our analysis (rs6107848) has been associated with BMI-adjusted waist circumference^27^. *RXRG* is a regulator of BMI-adjusted waist circumference^28^ and of QRS morphology^29^, QT interval^30^, appendicular lean mass^31^, gluteofemoral adipose tissue volumes^32^, and cardiovascular ageing^33^. The heritability of delta-BMI was estimated to be 0.109 (95% CI: 0.083–0.135, *p* ~ 0).

### Explainability: ECG morphologies associated with AI-ECG BMI predictions

**Fig. 9:**
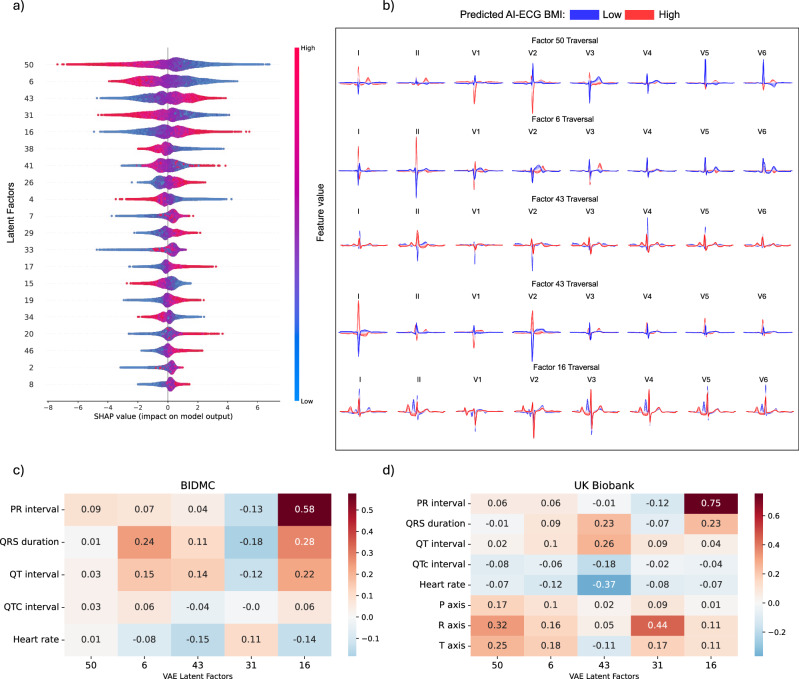
Explainable AI in ECG morphology. Explainable ECG morphology: An XGBoost model was trained using variational autoencoder-derived latent factors to estimate the AI-ECG-derived BMI predictions. **a** Depicts a beeswarm plot of the 20 most influential latent factors, ordered by their feature importance derived from the SHAP (SHapley Additive exPlanations) values. Each dot represents a SHAP value for a specific latent factor, providing insight into the significance of these latent factors and the direction of their impact on the AI-ECG BMI predictions. For example, for latent factor 50, lower values of the latent factor (in blue) indicate a positive impact on the AI-ECG BMI estimation, resulting in higher BMI predictions, while higher feature values (in red) indicate a negative impact on the AI-ECG BMI estimation, resulting in lower BMI predictions. **b** Illustrates the latent traversals of the top 5 latent features and their impact on the ECG morphology. ECG morphologies corresponding with high and low AI-ECG BMI predictions are represented in red and blue, respectively. Subplots **c** and **d** show correlation heatmaps between ECG parameters and the VAE-derived latent factors for the BIDMC and UK Biobank cohorts, respectively.

To enhance the interpretability of our AI-ECG BMI model, we trained an XGBoost model using variational autoencoder-derived latent factors to estimate the AI-ECG-derived BMI predictions. Figure 9a provides a visual representation highlighting the top 20 most important latent factors driving the AI-ECG BMI predictions of the XGBoost model. Figure 9b shows the latent traversals of the five most important latent factors in AI-ECG BMI predictions. To provide insight into the ECG morphologies associated with each latent factor, we conducted correlation analyses between the latent factors and ECG parameters in both the BIDMC and UK Biobank cohorts. Latent factors 50 and 6 are correlated with the QRS axis, latent factor 43 with heart rate, and 16 with PR interval duration. Additionally, QRS duration was found to be associated with multiple latent factors, including latent factors 6, 31, and 16 (Fig. 9c, d).

## Discussion

In this study, we trained an AI-ECG model to accurately identify an individual’s BMI. Importantly, we validated our model in two diverse populations, spanning secondary care and volunteers across two continents. Our findings highlight the model’s capacity to predict BMI across diverse populations. Notably, we evaluated the utility of delta-BMI as a predictor for future cardiometabolic disease, including type 2 diabetes mellitus, hypertension, and lipid disorders, demonstrating its potential prognostic value, which is additive to measured BMI. Additionally, we identified candidate biological pathways, including important phenotypic, genotypic, metabolomic, and proteomic associations. Using a variational autoencoder, we also provided insights into model prediction, which are in line with biologically plausible findings.

Using a secondary care dataset, we developed an AI-ECG BMI model that captures electrophysiological changes relating to adiposity. Furthermore, we externally validated the model in a cohort of healthy volunteers from the UK Biobank, underscoring its applicability in diverse population groups. Notably, the model performed well across both sexes and major ethnic groups. Interestingly, the model had improved performance in females compared to males, even though the derivation dataset had no significant differences in sex. The model may therefore be identifying obesity-related ECG changes that are more apparent in women than men.

Our model demonstrates better performance compared to Ryu et al.‘s BMI prediction model, with Pearson R of 0.65 (BIDMC) and 0.62 (UKB), and R^2^ values of 0.43 (BIDMC) and 0.39 (UKB), surpassing their reported R^2^ of 0.279. Despite their lower reported MAE of 2.332, the measured BMI of our cohorts exhibits markedly higher variance, enhancing the model’s applicability in clinical settings. Furthermore, our AI-ECG model compares favourably with other BMI models utilising more complex data. It aligns well with the brain MRI image-based model (Pearson *r* = 0.68)^34^, and though it exhibits lower correlation coefficients compared to models based on psychological variables (Pearson *r* = 0.81)^35^ and the UKB-PPP model using nearly 3000 protein variables (Pearson *r* = 0.89)^36^, its primary advantage lies in its accessibility. The widespread availability of ECG makes our AI-ECG model a practical and valuable tool for clinical settings.

Obesity is a major public health challenge, largely due to the associated risks of cardiometabolic disease. Although BMI is commonly used to define obesity, BMI itself poorly reflects the risk of cardiometabolic disease. Our findings demonstrate that delta-BMI yields additional insights beyond those provided by BMI alone, and an AI-ECG-predicted BMI exceeding the measured BMI may serve as an indicator of elevated risk for future cardiometabolic diseases.

Significant enhancements in model fit, reclassification, and minor but significant improvements in discriminatory power were observed with the inclusion of delta-BMI in both the BIDMC and UK Biobank cohorts. Notably, within the BIDMC cohort, notable enhancements in the concordance index were observed for patients with higher BMI (>25 and >30) when delta-BMI was added. This may be relevant for patients with metabolically healthy obesity^37^ – a subset of individuals with a BMI > 30, traditionally labelled as obese, who may exhibit a reduced risk of cardiometabolic disease. In this scenario, negative delta-BMI may serve as a valuable indicator, identifying those individuals with metabolically healthy obesity and offering a more nuanced and personalised assessment of risk.

Further analysis revealed that among the components of cardiometabolic disease, the most substantial improvement in model fit and net reclassification was observed for T2DM in both cohorts. Notable enhancements in discriminatory power were evident among patients with normal weight in the BIDMC cohort, whereas improvements spanned across all BMI categories in the UK Biobank cohort as well as female participants in both cohorts. This trend may reflect a broader effectiveness of the model in healthier populations, suggesting its potential utility for preventive healthcare interventions and risk assessment.

We also explored the additive effect of delta-BMI in various ethnic groups, finding significant improvements in model fit and reclassification for cardiometabolic disease in Caucasians and Hispanics, and for type 2 diabetes mellitus in Caucasians and Asians within the BIDMC cohort. However, due to small sample sizes within ethnic groups, further research in additional diverse datasets is necessary to evaluate the implications of these findings.

Subjects at risk of cardiometabolic disease already commonly have an ECG undertaken to identify prevalent cardiovascular diseases. However, in this work, we show the predictive capability of the ECG to predict future cardiometabolic disease, including hypertension, diabetes, and dyslipidaemia. This has important implications for screening of cardiometabolic disease, as the AI-ECG BMI model could be integrated into current practice to identify patients in need of increased surveillance, which may include blood pressure monitoring or more frequent blood sampling for HbA1c and lipids levels. Furthermore, delta-BMI could also act as a potential motivational tool for patients. Information about future risks, beyond that of simple BMI measurement, may help encourage lifestyle changes to improve cardiometabolic health.

The general reluctance to embrace AI tools in clinical settings often stems from the perceived “black box” nature of the models^38^. To enhance clinician and patient confidence in AI, it is crucial to prioritise explainability and biological plausibility. In our study, we have, through various methodologies, delved into the morphological and biological foundations of AI-ECG BMI predictions. This emphasis on transparency and biological plausibility aims not only to address the ‘black box’ concerns of AI but also to improve clinician and patient confidence in the application of AI-ECG-BMI in clinical settings.

Using a variational autoencoder model, we demonstrate that the QRS axis, resting heart rate, PR interval, and QRS duration are important in AI-ECG-BMI predictions. This is consistent with prior analyses relating the ECG to obesity-related cardiac changes^39–44^.

The ability of the AI-ECG BMI model to identify individuals at higher risk of future cardiometabolic disease raises important questions as to which clinical factors and biological pathways are identified in these predictions. Through a phenome-wide association study, we identified associations between delta-BMI and cardiometabolic conditions, including type 2 diabetes mellitus, hypertension, hyperlipidaemia, hypercholesterolaemia, liver disease, and atherosclerotic cardiovascular disease. Furthermore, delta-BMI is also associated with anthropometric measures of truncal obesity, such as abdominal fat ratio and waist circumference, aligning with previous studies highlighting the role of truncal obesity in cardiometabolic syndrome^45^ and mortality^46^. Interestingly, delta-BMI is also inversely associated with sex hormone-binding globulin (SHBG), which aligns with previous studies showing an inverse relationship between SHBG and adiposity^47,48^, coronary atherosclerosis, and cardiometabolic disease^49–51^. Furthermore, delta-BMI’s positive association with pericardial fat mass suggests that pericardial fat may play a role in mediating cardiometabolic risk, supported by its link to multiple measures of adiposity and metabolic syndrome^52,53^.

Through our metabolic profiling analyses, we have identified robust, independent metabolites associated with delta-BMI. Notably, these metabolites impact delta-BMI independently of each other and measured BMI. We observed significant negative associations between delta-BMI and glutamine, citrate, glycine, and cholesteryl esters in very large HDL. Conversely, metabolites such as omega-3 fatty acids, glucose, valine, total lipids in small HDL, and triglycerides in very large VLDL exhibited positive associations with delta-BMI. These findings are consistent with previous research indicating that glycine and glutamine are linked to improved insulin sensitivity, while valine is associated with reduced insulin secretion^54^. The positive link between omega-3 fatty acids and delta-BMI corresponds with evidence suggesting their detrimental impact on obesity, insulin resistance, and hyperlipidemia, despite their cardioprotective benefits in other metabolic conditions^55^. The observed inverse relationship between delta-BMI and cholesteryl esters in HDL, coupled with a positive correlation with total lipids in small HDL, suggests enhanced lecithin:cholesterol acyltransferase (LCAT) activity. This enzymatic action typically enriches HDL with lipids, while concurrently depleting its cholesteryl ester content, a metabolic signature noted in the presence of diabetes mellitus^56^. This is further corroborated by our proteomic profiling analysis, which identified LCAT as a stable protein positively associated with delta-BMI.

Through our plasma proteomic profiling analysis, we have identified robust, independent protein analytes associated with delta-BMI. We have observed negative associations between delta-BMI and CLPS, ADIPOQ, C9, HRC, and VCAN, and positive associations with STX3 and CNDP1. CLPS, implicated in the breakdown of dietary fats, is noted for genetic variations that are associated with metabolic characteristics in non-diabetic patients of European descent^57^. ADIPOQ is known to have decreased levels in conditions such as T2DM, obesity, and heart disease, implicating its importance in the metabolic homoeostasis^58^. STX3 is found to inhibit insulin release and production^59^, while CNDP1 is positively related to diabetic kidney disease and renal function markers^60^. The roles of C9, HRC, and VCAN in delta-BMI variability and cardiometabolic disease remain to be fully understood, highlighting a gap for further investigation.

Our study has limitations. Firstly, there was a temporal gap between the initial collection of the metabolomic and proteomic data in the UK Biobank and the subsequent acquisition of the resting ECGs, with the former occurring approximately 8 years before the latter. This temporal gap introduces noise in establishing relationships between the metabolomic, and proteomic markers and the electrocardiographic outcomes. Additionally, it’s important to note that the UK Biobank cohort is not ethnically diverse and exhibits a healthy volunteer bias, which may limit the generalisability of the findings to more diverse populations.

In conclusion, our study demonstrates the effectiveness of an AI-enhanced ECG model in accurately predicting BMI, validated across diverse populations. The introduction of delta-BMI as a predictor of cardiometabolic disease offers valuable additional prognostic insights beyond traditional BMI assessments. Our exploration of biological pathways, spanning phenotypic, genotypic, metabolomic, and proteomic associations, contributes to a comprehensive understanding of the association between delta-BMI and cardiometabolic disease. The clinical implications could extend to enhanced screening strategies, personalised risk assessment, and motivation for lifestyle interventions.

## Methods

### Ethical approvals

For the Beth Israel Deaconess Medical Center (BIDMC), cohort ethics review and approval were provided by the Beth Israel Deaconess Medical Center Committee on Clinical Investigations, IRB protocol # 2023P000042.

The UK Biobank has approval from the North West Multi-Centre Research Ethics Committee as a Research Tissue Bank (application IDs 48666, 47602).

### ECG datasets

As this was a retrospective study, no a priori sample size calculations were performed. Missing data was handled by complete-case analysis.

#### (i) The BIDMC cohort

The Beth Israel Deaconess Medical Center (BIDMC) cohort is a secondary care dataset consisting of routinely collected data from Boston, USA. Subjects over 16 years old with a valid ECG performed from 2014 to 2023 were included. Prior ECGs back to 2000 were included for these subjects. BMI was derived from contemporaneous weight measurements acquired within a 30-day window of each ECG recording, complemented by height assessments conducted within 1 year of the respective ECG. Diagnostic International Classification of Diseases (ICD) codes were used to determine disease status. Subjects were censored at the time of outcome or last in-person hospital contact.

#### (ii) The UK Biobank Cohort

The UK Biobank is a longitudinal study of over 500,000 volunteers aged 40–69 at the time of enrolment in 2006–2010^61^. At baseline assessment, participants provided information on health and lifestyle via questionnaire, had physical measures taken (including height, weight, and blood pressure), and donated samples of blood urine and saliva. A subgroup of participants was invited back for subsequent visits for additional investigations, including cardiac magnetic resonance imaging (MRI), brain MRI, and digital ECGs. 42,386 subjects with digital ECGs taken at the instance 2 visit were available for analysis. The collected data include clinical, metabolomic, proteomic, and genomic data, and were linked to cancer and death registries, hospital admissions, and primary care records. There is evidence of healthy volunteer selection bias^18^. Outcomes were linked to cancer and death registry data, hospital admissions, and primary care records. Detailed phenotyping using the cardiac MRI data has been previously described^62,63^.

### ECG pre-processing

The 12-lead ECGs from both cohorts were pre-processed using a bandpass filter at 0.5–100 Hz, a notch filter at 60 Hz, and re-sampled to 400 Hz. Zero padding was used to achieve a signal with 4096 samples for each lead for a 10 s recording.

### Model development

The model was derived using the BIDMC cohort. The BIDMC cohort consists of 1,163,401 ECGs from 189,540 patients, of which 512,950 (44.1%) ECGs from 114,415 subjects had paired BMI data available. To prevent data leakage, the dataset was divided by patient ID using a 60/10/30 split into derivation, validation, and holdout test sets, respectively. The ECG-AI model, which employed a ResNet architecture adapted from Ribeiro et al.^17^, was trained using 10-second 8-lead ECGs. Specifically, lead III and the augmented leads were omitted from the model as they are linear combinations of leads I and II. The architecture incorporates a linear function in the final layer for BMI prediction. The model was internally validated using the 30% holdout test set (152,166 ECGs from 34,325 patients).

### External validation

We externally validated the model using the UKB dataset. The original 42,386 ECGs were divided into validation and holdout test sets using a split ratio of 10/90%. The validation set was used to derive the bias-correction coefficients, which were then used to adjust the BMI estimates of the holdout test set. The holdout test set (*n* = 38,148) was used for subsequent downstream analyses.

### Outcome definition

BMI for the BIDMC cohort was calculated using patient height within a year and weight within 30 days of ECGs. For the UKB cohort, BMI was measured concurrently with ECGs. The primary survival analysis outcome was incident cardiometabolic disease. The secondary outcomes were incident type 2 diabetes, hypertension, and lipid disorders (see outcome definitions in Supplementary Table 3). Follow-up periods were adjusted by censoring at loss of follow-up or event occurrence.

### Bias correction

Delta-BMI was negatively correlated with BMI (Pearson *r* = −0.695, *p* ≈ 0.0), thus individuals with higher BMI demonstrated lower delta-BMI. This phenomenon, also known as the ‘regression dilution’ effect observed in previous models predicting biological age, underscores the need for bias correction to mitigate measurement error. Based on previous studies^33,64–66^, we addressed the correlation between delta-BMI and BMI in the holdout set by fitting a linear regression between the raw uncorrected delta-BMI and measured BMI in the validation dataset. Then, we adjusted the predicted BMI in the holdout set by subtracting the intercept and dividing it by the slope of the linear regression model^66^. This was repeated for the UKB cohort, using 10% of the dataset as a validation cohort to derive the regression model coefficients. This 10% validation cohort was excluded from subsequent downstream analyses. See Supplementary Fig. 1 for more details.

### Survival analysis and statistical analyses

The AI-ECG BMI model was assessed using Pearson correlation, R^2^, and MAE, with 95% CIs derived from 1000 bootstrap iterations on 80% of the holdout datasets. To evaluate the prognostic significance of delta-BMI in the BIDMC and UK Biobank cohorts, we conducted Cox regression, adjusting for BMI, age, and sex, and reported HRs and 95% CIs. Delta-BMI was analysed both as continuous and categorical variables. For the categorical analysis, delta-BMI was stratified into three tertiles: bottom (≤−3.74), middle (−3.74 to 2.44), and top (>2.44). Cox regression analyses were also conducted for stratified sex and BMI groups (18.5–24.9, ≥25, ≥30). Due to insufficient numbers, BMI < 18.5 was excluded (BIDMC: *n* = 794, UKB: *n* = 258). Consistent with recent evidence against the necessity of proportional hazards testing in well-powered clinical datasets, we did not assess this assumption, treating the Cox model’s hazard ratio as an average estimate over the follow-up period^67^.

Next, we performed an exploratory analysis to evaluate the incremental predictive utility of delta-BMI in predicting future cardiometabolic disease and its components. Firstly, we conducted likelihood ratio tests (LRT) to gauge the significance of model improvement upon incorporating delta-BMI. Secondly, we computed the continuous net reclassification index (NRI) to quantify the extent to which delta-BMI enhances risk stratification. Lastly, we examined changes in the concordance index upon the inclusion of delta-BMI into the Cox model, providing insights into its discriminatory power.

### Phenome-wide association study (PheWAS)

We conducted a phenome-wide association study (PheWAS) in the BIDMC cohort to determine delta-BMI’s disease associations, using univariate logistic regressions on 1408 disease phecodes. Similarly, in the UKB cohort, we analysed 1368 clinical phenotypes consisting of patient measurements, surveys and investigations, with delta-BMI, using univariate correlations. Adjustments were made for BMI, sex, age, and age^2^, as well as Bonferroni correction to account for multiple testing.

### Genome-wide association study (GWAS)

To identify genetic associations with delta-BMI, we performed a genome-wide association study (GWAS) in the UKB. Using linear regression, delta-BMI was adjusted for the following covariates: age at the imaging visit, sex, height, BMI, imaging assessment centre, and the first 10 genetic principal components. The UKB participants included in the genetic analyses were selected for European ancestry, missingness rate of SNPs <10%, no sex discrepancies, and outliers of heterozygosity or relatedness. After selection, ECGs were available for 27,988 individuals. Quality control was performed to exclude SNPs with a minor allele frequency <0.1%, genotyping rate <95%, deviation of heterozygosity with Hardy–Weinberg equilibrium *p* < 1.0 ×10^−8^ or <0.4 INFO imputation score.

The GWAS was carried out with the FastGWA MLM implemented by the Genome-wide Complex Trait Analysis (GCTA) software using a genetic relationship matrix (GRM) to adjust for population structure^68^. The delta-BMI distributions were normalised by rank-based inverse normal transform prior to the analysis. Age, sex, height, BMI, the UKB assessment centre, and the first 10 genetic principal components were included as covariates. We report the SNPs which were identified by the conventional genome-wide significance threshold of *p*-value < 5 ×10^−8^.

The genetic variance explained by genome-wide SNPs (SNP-based heritability) was calculated with the genomic-relatedness-based restricted maximum likelihood (GREML) analysis using the GCTA software^69^. Genetic correlation was calculated with the bivariate GREML analysis method^70^. The QQ plots for GWAS summary statistics and gene-based test are shown in Supplementary Fig. 5.

### Metabolome-wide association study (MWAS)

Over 250,000 UKB participants have been profiled by Nightingale Health to obtain their nuclear magnetic resonance (NMR) EDTA plasma sample metabolic biomarkers^71^. After extracting absolute measures and eliminating entries with over 40% missingness for rows and 20% for columns, we retained data for 274,350 participants from instance 0. We subsequently adjusted and standardised NMR metabolite readings for spectrometer variations and excluded outliers exceeding 4 interquartile ranges^71^. The final dataset, following filtering for complete cases, included 22,322 UK Biobank participants with both NMR metabolite profiles and 12-lead ECGs (UKB-NMRmet-ECG). To investigate the associations between delta-BMI and UKB NMR metabolites data, we initially conducted univariate correlation analyses of individual metabolites with delta-BMI, adjusted for BMI, sex, age, and age^2^, with a significance threshold of 2.976×10^−4^. Secondly, we employed stability selection with the least absolute shrinkage and selection operator (LASSO) regression to identify stable predictors of delta-BMI (R package Sharp, version 1.4.5^72^). The stability selection with LASSO was conducted over 1000 iterations with subsampling of 80% of the total UKB-NMRmet-ECG dataset, with lambda constrained to 1 ×10^−8^ and 5 ×10^−^^1^. The calibration of *π* (selection proportion) and *λ* (L1 penalty factor) is shown in Supplementary Fig. 2. The combination of parameters resulting in the highest stability score was selected (*π* = 0.650 and *λ* = 0.142), thus obtaining 14 stably selected predictors. These predictors were then used in a multivariate linear regression to disentangle the metabolomic associations with delta-BMI, as seen in Fig. 6c.

### Proteomic-wide association study (PWAS)

53,030 UKB participants had their plasma proteomic profiles analysed by the Pharma Proteomics Project^36^ at instance 0 using the antibody-based Olink Explore 3072 PEA, measuring 2923 protein analytes^36^. Observations with less than 40% missingness and protein analytes with less than 20% missingness were retained, leaving 2919 analytes for further analysis. Missing values were imputed using simple mean imputation and scaled (sci-kit-learn, version 1.1.1) (Supplementary Fig. 3). In total, 3512 patients had both proteomic data and 12-lead ECGs (UKB-PPP-ECG). To investigate the associations between delta-BMI and the UK Biobank plasma proteomic data, we mirrored the metabolomic approach. Initially, we conducted a univariate regression analysis with delta-BMI as outcome and protein analytes as predictors, adjusted for BMI, sex, age, and age^2^, with a significance threshold of 1.7123 ×10^−5^. Secondly, we performed stability selection with LASSO regression to identify stably selected protein predictors of delta-BMI. Variable selection using the Sharp package was done over 1000 iterations with a subsampling of 80% of the total UKB-PPP-ECG dataset. The calibration of *π* and *λ* is depicted in Supplementary Fig. 4. The combination of parameters with the highest stability score was selected (*π* = 0.560 and *λ* = 0.107), thus identifying 39 stably selected predictors. The stable predictors were then used in multivariate linear regression to disentangle the proteomic associations with delta-BMI, as seen in Fig. 7c.

### Explainability

To understand the ECG morphologies associated with the AI-ECG BMI predictions, we trained a VAE, as previously described^73^, using median ECG beats. Median beats were extracted using the BRAVEHEART software, as previously described^74^. The VAE was based on a convolutional encoder/decoder architecture, which was inspired by architectures previously used for ECG analysis^75,76^. Specifically, the encoder comprised of six convolutional blocks of feature extraction, adjusted for the median beat ECG signal. The decoder architecture was designed as a symmetrically inverse network of the encoder. The total number of parameters was 1,533,888 and 1,283,976 for the encoder and decoder, respectively. A detailed depiction of the networks’ architecture can be seen in Supplementary Fig. 6.

Using the same data split as for the AI-ECG BMI model, we then trained an eXtreme Gradient Boosting (XGBoost) model using the VAE latent features with AI-ECG BMI as the output. In the BIDMC holdout set, the XGBoost model achieved a Pearson correlation coefficient of 0.77 (95% CI: 0.77–0.78) and R^2^ of 0.61 (95% CI: 0.60–0.61) (Supplementary Fig. 7a). The model was then validated in the UK Biobank, where it achieved a Pearson correlation coefficient of 0.78 (95% CI: 0.78–0.79) and R^2^ of 0.61 (95% CI: 0.600–0.62) (Supplementary Fig. 7b). The top 5 most important features, as assessed by SHAP values (Fig. 9a), were visualised by latent traversals^73^ (Fig. 9b) and cross-correlated with known ECG parameters (Fig. 9c, d).

### Reporting summary

Further information on research design is available in the Nature Research Reporting Summary linked to this article.

### Supplementary information

Supplementary material

Online supplement

Reporting Summary

## Data Availability

UK Biobank data are available upon application (http://www.ukbiobank.ac.uk/). The study was conducted under application numbers 48666 and 47602. The GWAS summary level data are available from the GWAS catalogue (https://ebi.ac.uk/gwas/) through the accession number GCST90429062. The BIDMC dataset is restricted due to ethical limitations. Researchers affiliated to educational, or research institutions may make requests to access the datasets. Requests should be made to the corresponding author of this paper. They will be forwarded to the relevant steering committee.

## Code availability

The programming code relating to these analyses will be made available under GNU General Public License version 3 upon request to the corresponding author.
