# Supplementary material for: Artificial intelligence-enhanced electrocardiography derived body mass index as a predictor of future cardiometabolic disease
Source: NPJ Digit Med. 2024 Jun 25;7:167. doi: 10.1038/s41746-024-01170-0 (PMC11199586; doi:10.1038/s41746-024-01170-0)
Supplement: Supplementary file 1 — Supplementary material [file 41746_2024_1170_MOESM1_ESM.pdf]

Artificial Intelligence-enhanced Electrocardiography Derived Body Mass Index as a Predictor of Future Cardiometabolic Disease

Supplement

Table of Contents

Supplementary Table 1: Cohort demographics 3

Supplementary Table 2: Model performance 4

Supplementary Table 3: Outcome definition 5

Supplementary Figure 1: Model predictions and bias correction 5

Supplementary Table 4: Survival analysis – continuous outcomes 8

Supplementary Table 5: Model improvement 10

Supplementary Figure 2 – Metabolome: calibration plot of stability selection with LASSO 12

Supplementary Figure 3: Pre-processing steps for the UK Biobank Proteomic and ECG dataset (UKB-PPP-ECG) 13

Supplementary Figure 4: Proteome: calibration plot of stability selection with LASSO 14

Supplementary Figure 5: GWAS QQ plots 15

Supplementary Figure 6: The VAE Convolutional Architecture 16

Supplementary Figure 7: AI-ECG BMI model explainability 17

Supplementary Table 1: Cohort demographics

Summary of Patient Characteristics in the BIDMC and UK Biobank Cohorts. The table presents key demographic and clinical attributes, including age, gender distribution, BMI, blood pressure measurements, days to censor, prevalence of cardiometabolic disease, T2DM, hypertension, and lipid disorders, and ethnic distribution.

		BIDMC	UK Biobank
**Total, n**		512950	42386
**Age, median [Q1,Q3]**		66.5 [55.4,76.4]	65.0 [58.0,70.0]
**Female, n (%)**		255787 (49.9)	21848 (51.5)
**BMI, median [Q1,Q3]**		27.9 [24.3,32.5]	25.9 [23.5,28.9]
**Systolic BP, mean (SD)**		129.2 (14.1)	141.1 (19.9)
**Diastolic BP, mean (SD)**		72.9 (9.4)	78.9 (10.6)
**Days to censor, median [Q1,Q3]**		1380 [505,2344]	1674 [1355,2219]
**Cardiometabolic Disease, n (%)**		64455 (12.6)	2759 (6.5)
**T2DM, n (%)**		49884 (9.7)	528 (1.2)
**Hypertension n (%)**		61632 (12.0)	6798 (16.0)
**Lipid Disorders, n (%)**		71887 (14.0)	2183 (5.2)
**Ethnicity, n (%)**	Caucasian	347935 (67.8)	40926 (96.6)
	Asian	18697 (3.6)	603 (1.4)
	African American/Black or British	81369 (15.9)	305 (0.7)
	Hispanic	31890 (6.2)	N/A
	Other	18246 (3.6)	436 (1.0)
	Unknown/Declined	14813 (2.9)	103 (0.2)

Supplementary Table 2: Model performance

Performance Metrics of the Predictive Model for Body Mass Index (BMI) Estimation. The table displays the Mean Absolute Error (MAE) of raw and bias-corrected predictions, Pearson correlation coefficient (Pearson r), and coefficient of determination (R^2) for BMI predictions across subgroups in the BIDMC and UK Biobank cohorts. These metrics are accompanied by 95% confidence intervals. Results are presented for the overall cohort as well as stratified by gender and ethnicity, providing insights into the model's accuracy and performance within diverse population segments.

		n (%)	Raw MAE (95% CI)	Bias-corrected MAE (95% CI)	Pearson r (95% CI)	R^2 (95% CI)
**BIDMC**	**All**	152166 (100)	3.95 (3.93-3.97)	6.14 (6.11-6.17)	0.65 (0.65-0.66)	0.43 (0.42-0.43)
	**Female**	75,523 (49.6)	4.21 (4.18-4.24)	6.54 (6.49-6.58)	0.68 (0.67-0.68)	0.46 (0.45-0.47)
	**Male**	76,643 (50.4)	3.69 (3.67-3.72)	5.75 (5.70-5.79)	0.62 (0.61-0.63)	0.39 (0.38-0.39)
	**Caucasian**	102,429 (67.3)	3.88 (3.85-3.90)	6.03 (5.99-6.07)	0.65 (0.64-0.65)	0.42 (0.41-0.42)
	**African-American**	24,572 (16.2)	4.33 (4.27-4.39)	6.75 (6.67-6.83)	0.68 (0.66-0.69)	0.46 (0.44-0.47)
	**Asian**	5,758 (3.8)	3.57 (3.50-3.65)	5.49 (5.37-5.62)	0.58 (0.56-0.60)	0.34 (0.31-0.36)
	**Hispanic**	9,608 (6.3)	4.01 (3.94-4.08)	6.23 (6.11-6.33)	0.61 (0.60-0.63)	0.37 (0.36-0.39)
**UK Biobank**	**All**	38,148 (100)	2.94 (2.91-2.96)	4.38 (4.34-4.42)	0.62 (0.62-0.63)	0.39 (0.38-0.40)
	**Female**	19,691 (51.6)	3.05 (3.01-3.09)	4.70 (4.64-4.76)	0.64 (0.63-0.65)	0.40 (0.39-0.42)
	**Male**	18,455 (48.4)	2.82 (2.78-2.85)	4.04 (3.98-4.10)	0.59 (0.58-0.61)	0.35 (0.34-0.37)
	**Caucasian**	36,827 (96.5)	2.93 (2.90-2.95)	4.37 (4.33-4.41)	0.62 (0.62-0.63)	0.39 (0.38-0.40)
	**Black or Black British**	278 (0.7)	3.10 (2.81-3.42)	5.00 (4.45-5.58)	0.59 (0.47-0.69)	0.35 (0.22-0.48)
	**Asian or Asian British**	435 (1.1)	3.36 (3.11-3.61)	4.53 (4.11-4.94)	0.57 (0.48-0.65)	0.33 (0.23-0.42)
	**Mixed**	183 (0.5)	3.11 (2.70-3.50)	4.32 (3.66-5.03)	0.68 (0.55-0.78)	0.47 (0.30-0.61)

Supplementary Table 3: Outcome definition

Primary and Secondary Outcome Definitions with Corresponding ICD-9 and ICD-10 Codes for Cardiometabolic Disease, Type 2 Diabetes Mellitus, Hypertension, and Lipid Disorders in the Study Cohort.

Outcome	ICD9	ICD10
**Cardiometabolic disease**	250.00, 250.02, 250.9, 250.92, 250.1, 250.12, 250.2, 250.22, 250.3, 250.32 250.40, 250.50, 250.52, 250.60, 250.70, 250.72, 250.80, 250.82, 250.90, 250.92	E11, E13, E14, G59<br>I10<br>E78
**Type 2 diabetes mellitus**	250.00, 250.02, 250.9, 250.92, 250.1, 250.12, 250.2, 250.22, 250.3, 250.32 250.40, 250.50, 250.52, 250.60, 250.70, 250.72, 250.80, 250.82, 250.90, 250.92	E11, E13, E14, G59
**Hypertension**	401, 402, 403, 404, 405	I10
**Lipid Disorders**	272	E78

Supplementary Figure 1: Model predictions and bias correction

A-B) Scatter plots depicting the association between bias-corrected AI-ECG-BMI and BMI within the 30% holdout BIDMC and UK Biobank cohorts. The solid black identity line serves as a reference point, representing the ideal prediction scenario. The red line represents the best fit line. C-D) Scatter plots demonstrating the relationship between bias-corrected AI-ECG delta BMI and BMI in the 30% holdout BIDMC and UK Biobank cohorts. A horizontal black line is included in these panels to serve as a reference, indicating the absence of a relationship between delta BMI and BMI. E-F) Bland-Altman plots for the BIDMC (30% holdout) and UK Biobank cohorts illustrate the comparison between bias-corrected AI-ECG-BMI predictions and measured BMI. The red line denotes the mean difference, while the two horizontal dashed black lines signify the upper and lower 95% confidence intervals. In both cohorts, the Bland-Altman analysis reveals that the mean difference falls within a narrow range

around zero, indicating a balanced agreement between bias-corrected AI-ECG-BMI predictions and measured BMI. This consistency suggests the absence of systemic bias.

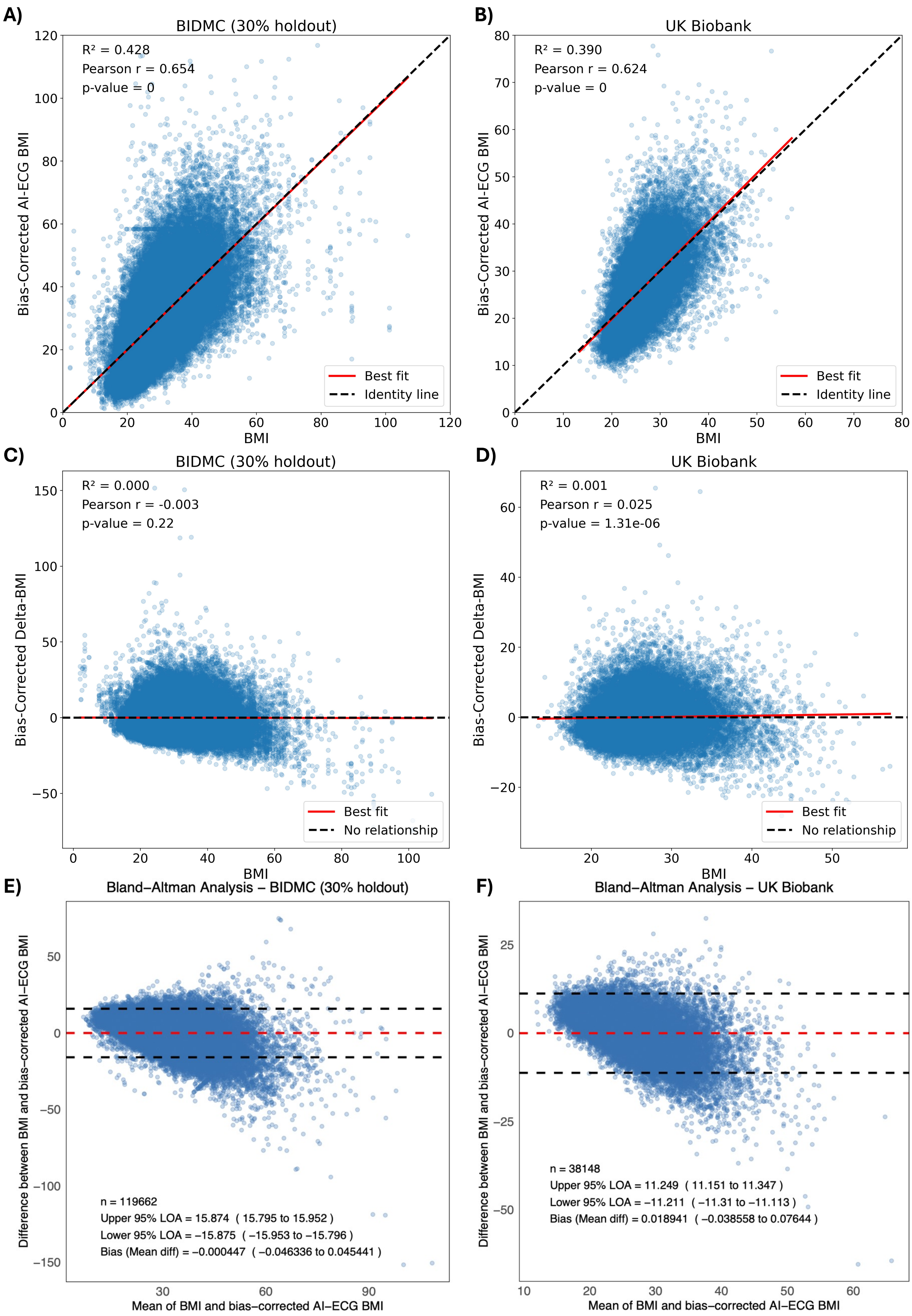
A)
BIDMC (30% holdout)
R² = 0.428
Pearson r = 0.654
p-value = 0
Bias-Corrected AI-ECG BMI
BMI
Best fit
Identity line
B)
UK Biobank
R² = 0.390
Pearson r = 0.624
p-value = 0
Bias-Corrected AI-ECG BMI
BMI
Best fit
Identity line
C)
BIDMC (30% holdout)
R² = 0.000
Pearson r = -0.003
p-value = 0.22
Bias-Corrected Delta-BMI
BMI
Best fit
No relationship
D)
UK Biobank
R² = 0.001
Pearson r = 0.025
p-value = 1.31e-06
Bias-Corrected Delta-BMI
BMI
Best fit
No relationship
E)
Bland–Altman Analysis – BIDMC (30% holdout)
Difference between BMI and bias–corrected AI–ECG BMI
n = 119662
Upper 95% LOA = 15.874 (15.795 to 15.952)
Lower 95% LOA = –15.875 (–15.953 to –15.796)
Bias (Mean diff) = –0.000447 (–0.046336 to 0.045441)
Mean of BMI and bias–corrected AI–ECG BMI
F)
Bland–Altman Analysis – UK Biobank
Difference between BMI and bias–corrected AI–ECG BMI
n = 38148
Upper 95% LOA = 11.249 (11.151 to 11.347)
Lower 95% LOA = –11.211 (–11.31 to –11.113)
Bias (Mean diff) = 0.018941 (–0.038558 to 0.07644)
Mean of BMI and bias–corrected AI–ECG BMI

Supplementary Table 4: Survival analysis – continuous outcomes

Hazard Ratios from survival analysis of continuous AI-ECG delta BMI adjusted for measured BMI, age, and sex, on the future incidence of Cardiometabolic Disease, type 2 diabetes mellitus, hypertension, and lipid disorders in the BIDMC holdout set and the UK Biobank cohort. Sub-analyses include Cardiometabolic Disease stratified by BMI categories (18.5 – 24.9, ≥ 25, ≥30), as well as by sex for both the BIDMC holdout set and the UK Biobank cohort.

BIDMC (holdout)							
**Disease**	**Delta BMI**	**95% CI**	**p-value**	**z**	**Age**	**Sex**	**BMI**
**Cardiometabolic disease**	1.01	1.00 – 1.01	<0.001	4.7	1.02	0.83	1.02
**Type 2 Diabetes Mellitus**	1.01	1.01 – 1.02	<0.001	11.1	1.01	0.71	1.06
**Hypertension**	1.01	1.01 – 1.02	<0.001	9.3	1.02	0.77	1.03
**Lipid Disorders**	1.01	1.01 – 1.01	<0.001	9.5	1.02	0.78	1.03
**BIDMC (holdout) – Cardiometabolic disease**							
**Outpatient, BMI 18.5 – 24.9**	1.02	1.01 – 1.03	<0.001	4.1	1.03	0.82	1.04
**Outpatient, BMI > 25**	1.01	1.01 – 1.02	<0.001	4.0	1.01	0.97	1.00
**Outpatient, BMI > 30**	1.01	1.00 – 1.02	0.002	3.1	1.00	0.99	1.01
**Outpatient, Females**	1.02	1.01 – 1.02	<0.001	5.2	1.02	N/A	1.02
**Outpatient, Males**	1.01	1.00 – 1.02	0.003	2.9	1.01	N/A	1.01
**UK Biobank**							
**Cardiometabolic disease**	1.02	1.02 – 1.03	<0.005	7.2	1.06	0.71	1.09
**Type 2 Diabetes Mellitus**	1.05	1.04 – 1.07	<0.005	8.3	1.04	0.50	1.15
**Hypertension**	1.02	1.02 – 1.03	<0.005	6.0	1.06	0.71	1.10
**Lipid Disorders**	1.02	1.01 – 1.03	<0.005	3.9	1.06	0.60	1.06
**UK Biobank – Cardiometabolic Disease**							
**BMI 18.5 – 24.9**	1.03	1.01 – 1.04	<0.005	3.8	1.08	0.80	1.12
**BMI > 25**	1.02	1.02 – 1.03	<0.005	5.8	1.05	0.68	1.08
**BMI > 30**	1.03	1.02 – 1.04	<0.005	4.6	1.05	0.68	1.07
**Females**	1.03	1.02 – 1.04	<0.005	6.0	1.07	N/A	1.09
**Males**	1.02	1.01 – 1.03	<0.005	4.1	1.06	N/A	1.09

Supplementary Table 5: Model improvement

Analysis of model improvement with delta-BMI, evaluated through likelihood ratio tests (LRT), continuous net reclassification index (NRI), and differences in concordance index (Δ C-index) across both the BIDMC and UK Biobank cohorts and their respective subgroups.

BIDMC (holdout)						
**Type**	**LRT: χ^2**	**p-value**	**NRI**	**95% CI**	**Δ C-index**	**95% CI**
**Cardiometabolic disease**	21.9	< 0.0001	0.097	0.056 - 0.129	0.0055	0.0015 - 0.0091
**Type 2 Diabetes Mellitus**	118.1	< 0.0001	0.172	0.125 - 0.213	0.0022	0.0008 - 0.0035
**Hypertension**	83.1	< 0.0001	0.125	0.093 - 0.155	0.0035	0.0018 - 0.0052
**Lipid Disorders**	84.8	< 0.0001	0.132	0.104 - 0.162	0.0018	0.0004 - 0.0030
**UK Biobank**						
**Cardiometabolic disease**	36.6	< 0.0001	0.161	0.117 - 0.200	0.0038	0.0014 - 0.0059
**Type 2 Diabetes Mellitus**	44.9	< 0.0001	0.323	0.230 - 0.433	0.0121	0.0049 – 0.0195
**Hypertension**	26.2	< 0.0001	0.145	0.102 - 0.192	0.0029	0.0007 - 0.0049
**Lipid Disorders**	12.6	0.00039	0.102	0.041 - 0.166	0.0025	-0.0002 - 0.0048
**BIDMC (holdout) – Cardiometabolic Disease**						
**Outpatient, BMI 18.5 – 24.9**	16.0	< 0.0001	0.109	0.016 - 0.189	0.0027	-0.0016 - 0.0064
**Outpatient, BMI > 25**	14.9	0.00012	0.090	0.029 - 0.161	0.0098	0.0020 – 0.0177
**Outpatient, BMI > 30**	8.9	0.0029	0.108	0.009 - 0.224	0.0362	0.0218 – 0.0755
**Outpatient, Females**	23.8	< 0.0001	0.133	0.060 - 0.198	0.0051	0.0008 - 0.0091
**Outpatient, Males**	8.3	0.0039	0.100	0.008 - 0.169	0.0074	-0.0022 – 0.0163
**Outpatient, Caucasian**	21.5	< 0.0001	0.114	0.045 - 0.168	0.0086	0.0031 – 0.0146
**Outpatients, African American**	0.4	0.53	0.057	-0.115 - 0.219	-0.0004	-0.0060 - 0.0038
**Outpatients, Asian**	0.03	0.86	0.070	-0.156 - 0.363	0.0157	-0.0029 – 0.0424
**Outpatients, Hispanic**	11.8	0.00059	0.287	0.091 - 0.505	0.0173	-0.0069 – 0.0417
**BIDMC (holdout) – T2DM**						
**Outpatient, BMI 18.5 – 24.9**	42.0	< 0.0001	0.366	0.261 - 0.452	0.0239	0.0053 – 0.0377
**Outpatient, BMI > 25**	36.7	< 0.0001	0.092	0.048 - 0.133	0.0029	0.0004 - 0.0053
**Outpatient, BMI > 30**	3.8	0.0523	0.028	-0.024 - 0.082	0.0001	-0.0018 - 0.0015
**Outpatient, Females**	80.9	< 0.0001	0.222	0.163 - 0.277	0.0108	0.0056 – 0.0162
**Outpatient, Males**	7.4	0.0064	0.079	0.025 - 0.128	-0.0002	-0.0012 - 0.0007
**Outpatient, Caucasian**	37.5	< 0.0001	0.158	0.108 - 0.203	0.0024	0.0003 – 0.0044
**Outpatients, African American**	3.9	0.048	0.038	-0.047 - 0.146	0.0004	-0.0031 – 0.0033
**Outpatients, Asian**	4.8	0.029	0.265	0.052 - 0.462	0.0117	-0.0077 – 0.0304
**Outpatients, Hispanic**	19.4	< 0.0001	0.099	-0.022 - 0.266	0.0097	0.0008 – 0.0186
**UK Biobank – Cardiometabolic Disease**						
**BMI 18.5 – 24.9**	10.0	0.0016	0.094	0.014 - 0.179	0.0041	-0.0006 - 0.0084
**BMI > 25**	24.0	< 0.0001	0.094	0.016 - 0.180	0.0044	0.0012 - 0.0076
**BMI > 30**	14.1	0.00018	0.125	0.039 - 0.236	0.0071	-0.0009 - 0.0043
**Females**	27.4	< 0.0001	0.180	0.125 - 0.259	0.0063	0.0017 – 0.0105
**Males**	11.1	0.00085	0.121	0.051 - 0.179	0.0015	-0.0009 - 0.0037
**UK Biobank – T2DM**						
**BMI 18.5 – 24.9**	14.9	0.00012	0.220	0.021 - 0.464	0.0377	0.0036 – 0.0751
**BMI > 25**	37.0	< 0.0001	0.285	0.168 - 0.379	0.0186	0.0080 – 0.0300
**BMI > 30**	20.3	< 0.0001	0.251	0.104 - 0.413	0.0299	0.0055 – 0.0546
**Females**	28.2	< 0.0001	0.436	0.262 - 0.590	0.0153	0.0011 – 0.0297
**Males**	19.1	< 0.0001	0.246	0.126 - 0.366	0.0122	0.0017 – 0.0227

Supplementary Figure 2 – Metabolome: calibration plot of stability selection with LASSO

Stability Selection (LASSO) applied on the UKB-NMRmet-ECG dataset with delta-BMI as the outcome. The calibration plot shows the stability scores for each iteration, with the highest stability score at π = 0.650 and λ = 0.142, thus obtaining 14 stably selected predictors.

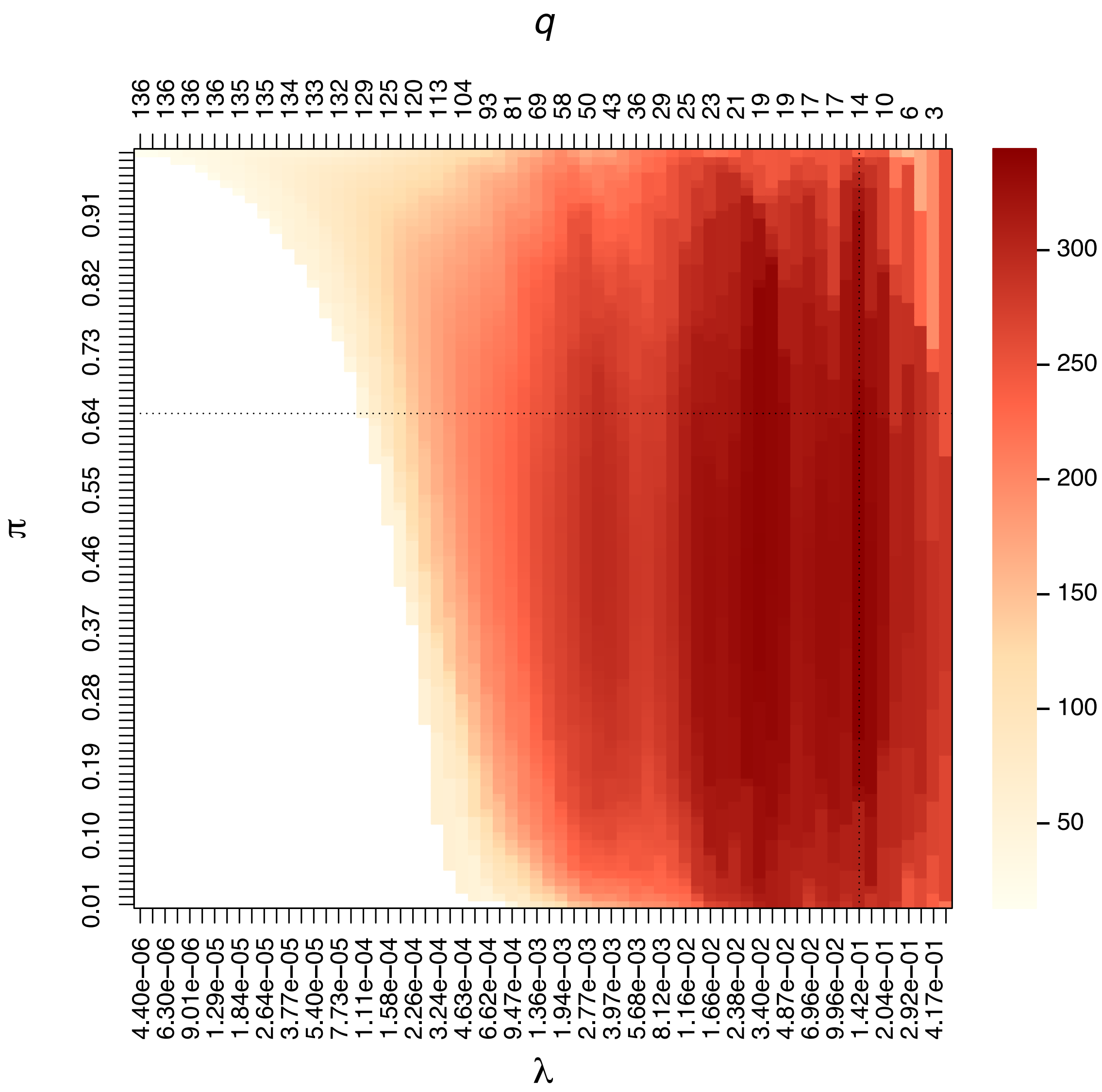

Supplementary Figure 3: Pre-processing steps for the UK Biobank Proteomic and ECG dataset (UKB-PPP-ECG)

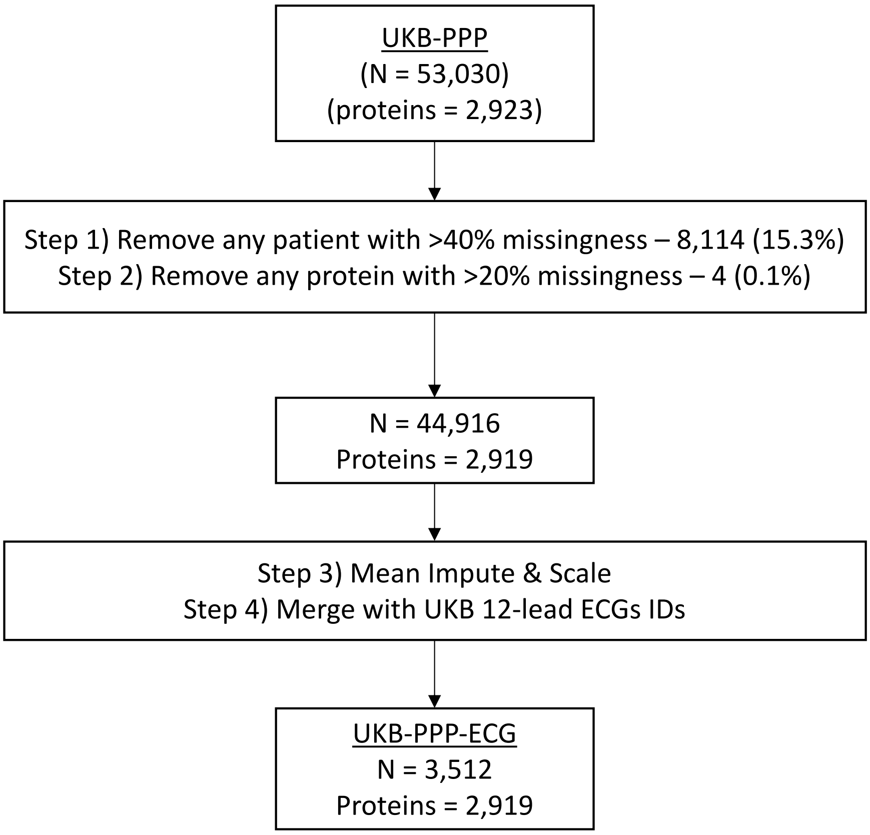

Supplementary Figure 4: Proteome: calibration plot of stability selection with LASSO

Stability Selection (LASSO) applied on the UKB-PPP-ECG dataset with delta-BMI as the outcome. The calibration plot shows the stability scores for each iteration, with the highest stability score at π = 0.560 and λ = 0.107, thus obtaining 39 stably selected variables.

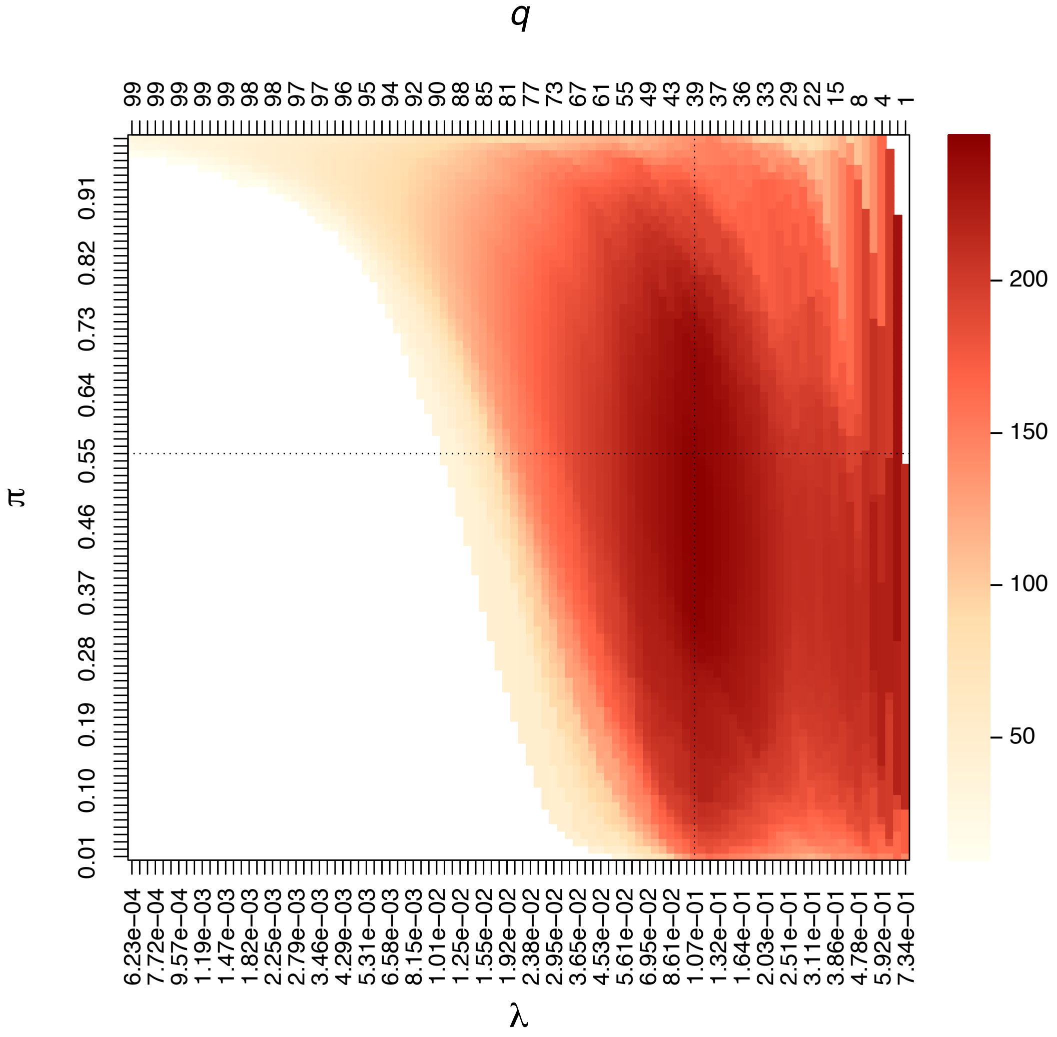

Supplementary Figure 5: GWAS QQ plots

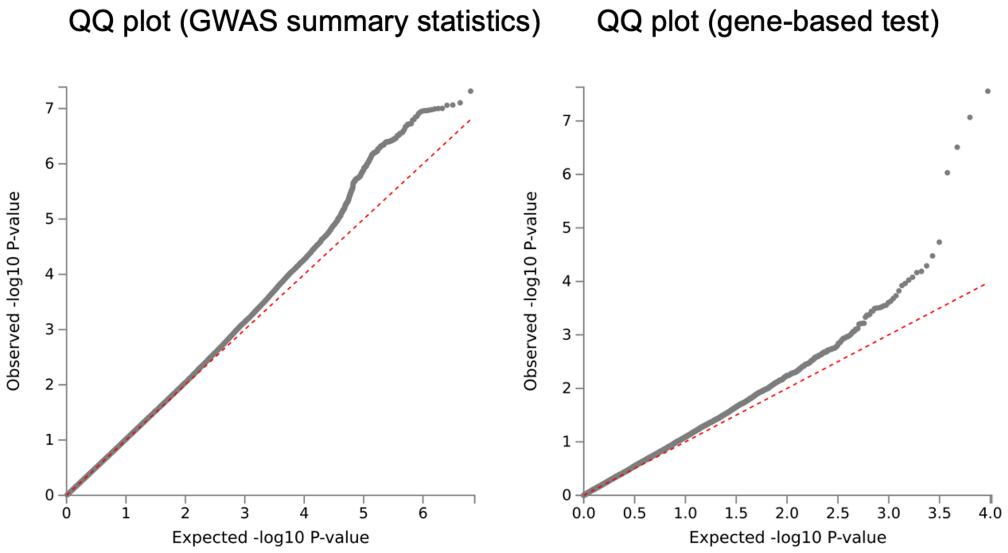

i. GC lambda 0.5: 1.021
GC lambda 0.1: 1.023
GC lambda 0.01: 1.027
GC lambda 0.001: 1.055

Supplementary Figure 6: The VAE Convolutional Architecture

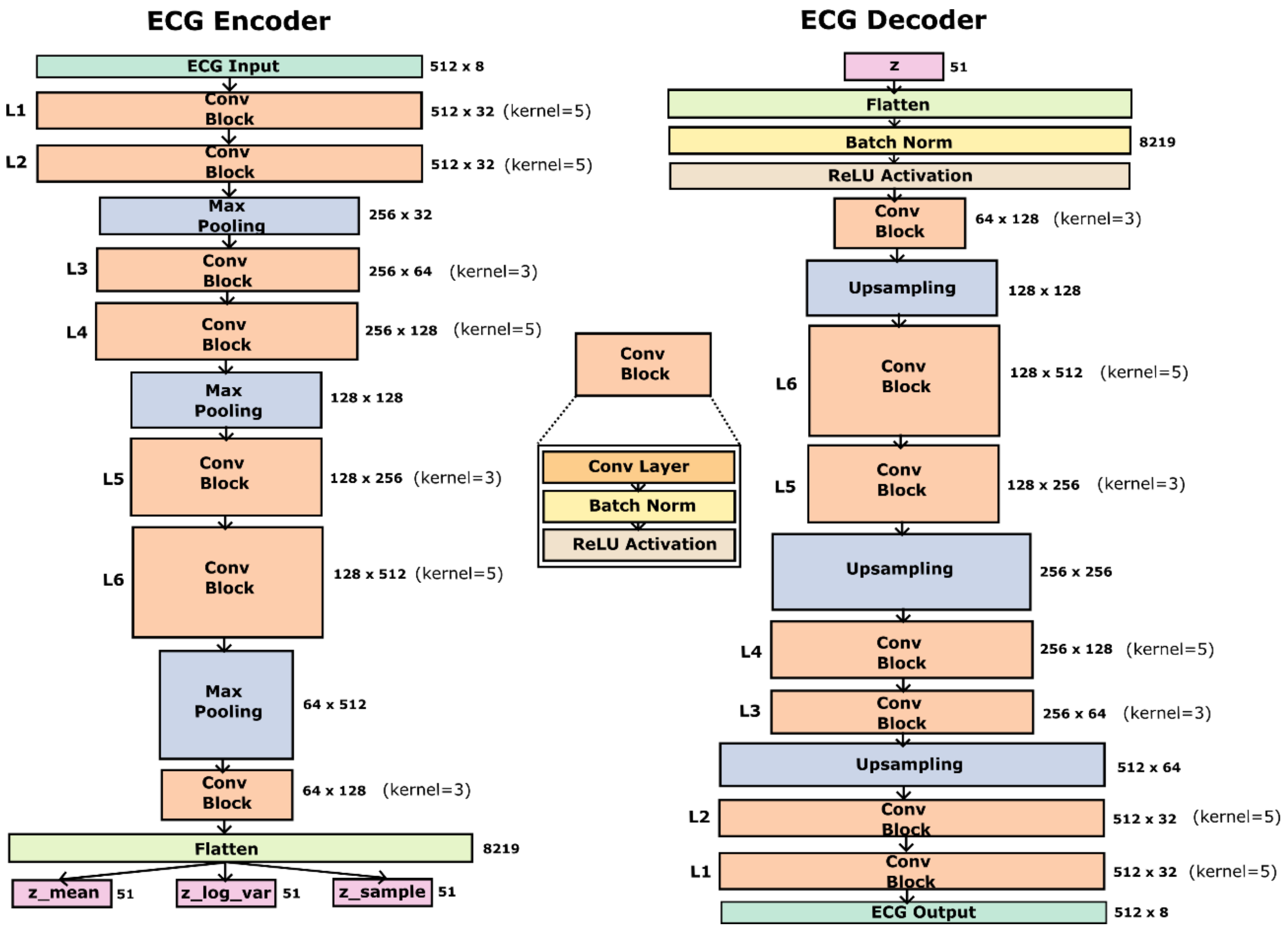

Supplementary Figure 7: AI-ECG BMI model explainability

A variational auto-encoder-derived latent factors were used in an explainable XGBoost model to predict AI-ECG BMI predictions in A) the BIDMC cohort, and B) the UK Biobank cohort.

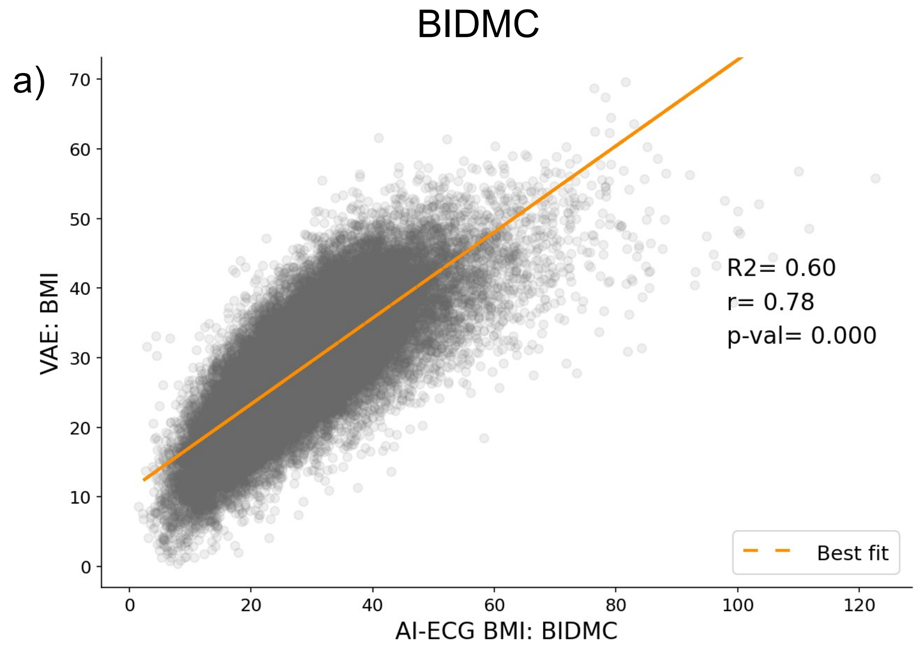
BIDMC
a)
R2= 0.60
r= 0.78
p-val= 0.000
Best fit
VAE: BMI
AI-ECG BMI: BIDMC

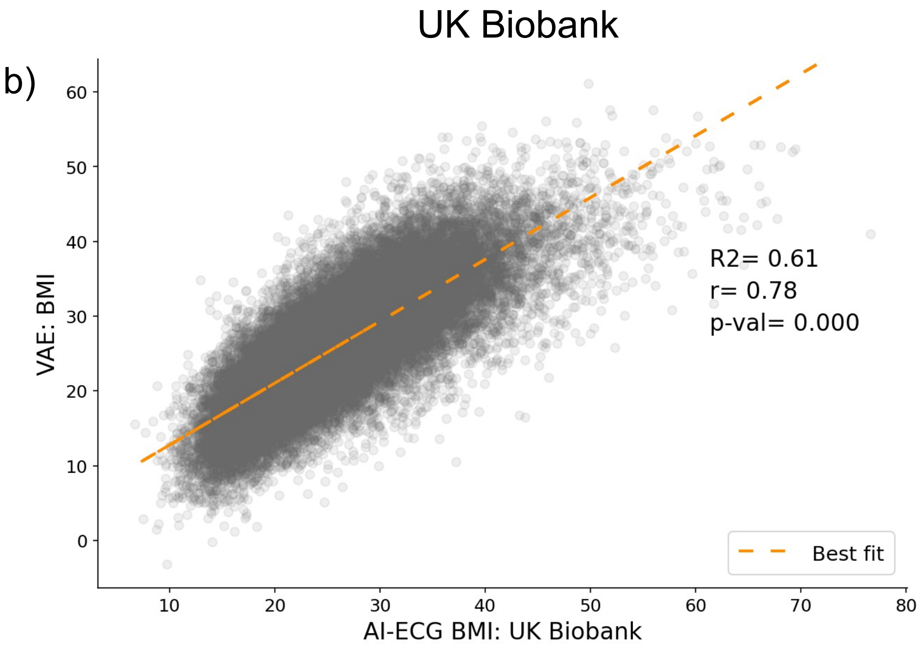
UK Biobank
b)
R2= 0.61
r= 0.78
p-val= 0.000
Best fit
VAE: BMI
AI-ECG BMI: UK Biobank
