# Supplementary material for: Artificial intelligence-enhanced electrocardiography derived body mass index as a predictor of future cardiometabolic disease
Source: NPJ Digit Med. 2024 Jun 25;7:167. doi: 10.1038/s41746-024-01170-0 (PMC11199586; doi:10.1038/s41746-024-01170-0)
Supplement: Supplementary file 3 — Reporting Summary [file 41746_2024_1170_MOESM3_ESM.pdf]

Corresponding author(s): Libor Pastika
Fu Siong Ng

Last updated by author(s): 29/05/24

Reporting Summary

Nature Portfolio wishes to improve the reproducibility of the work that we publish. This form provides structure for consistency and transparency in reporting. For further information on Nature Portfolio policies, see our Editorial Policies and the Editorial Policy Checklist.

Statistics

For all statistical analyses, confirm that the following items are present in the figure legend, table legend, main text, or Methods section.

n/a	Confirmed	
☐	☒	The exact sample size (*n*) for each experimental group/condition, given as a discrete number and unit of measurement
☐	☒	A statement on whether measurements were taken from distinct samples or whether the same sample was measured repeatedly
☐	☒	The statistical test(s) used AND whether they are one- or two-sided<br>*Only common tests should be described solely by name; describe more complex techniques in the Methods section.*
☐	☒	A description of all covariates tested
☐	☒	A description of any assumptions or corrections, such as tests of normality and adjustment for multiple comparisons
☐	☒	A full description of the statistical parameters including central tendency (e.g. means) or other basic estimates (e.g. regression coefficient) AND variation (e.g. standard deviation) or associated estimates of uncertainty (e.g. confidence intervals)
☐	☒	For null hypothesis testing, the test statistic (e.g. *F*, *t*, *r*) with confidence intervals, effect sizes, degrees of freedom and *P* value noted<br>*Give P values as exact values whenever suitable.*
☒	☐	For Bayesian analysis, information on the choice of priors and Markov chain Monte Carlo settings
☒	☐	For hierarchical and complex designs, identification of the appropriate level for tests and full reporting of outcomes
☐	☒	Estimates of effect sizes (e.g. Cohen's *d*, Pearson's *r*), indicating how they were calculated

Our web collection on statistics for biologists contains articles on many of the points above.

Software and code

Policy information about availability of computer code

Data collection	No specific software used for data collection
Data analysis	Python version 3.9, R 4.0.0

For manuscripts utilizing custom algorithms or software that are central to the research but not yet described in published literature, software must be made available to editors and reviewers. We strongly encourage code deposition in a community repository (e.g. GitHub). See the Nature Portfolio guidelines for submitting code & software for further information.

Data

Policy information about availability of data

All manuscripts must include a data availability statement. This statement should provide the following information, where applicable:

- Accession codes, unique identifiers, or web links for publicly available datasets
- A description of any restrictions on data availability
- For clinical datasets or third party data, please ensure that the statement adheres to our policy

UK Biobank data are available upon application (http://www.ukbiobank.ac.uk/). The BIDMC dataset is restricted due to ethical limitations. Researchers affiliated to educational, or research institutions may make requests to access the datasets. Requests should be made to the corresponding author of this paper. They will be forwarded to the relevant steering committee.

Research involving human participants, their data, or biological material

Policy information about studies with human participants or human data. See also policy information about sex, gender (identity/presentation), and sexual orientation and race, ethnicity and racism.

Reporting on sex and gender	Analyses were adjusted for self-reported sex, excluding GWAS, which used genetically determined sex. Sample sizes are provided in the manuscript.
Reporting on race, ethnicity, or other socially relevant groupings	Supplementary Table 1 provides a summary of the ethnicities of the participants. Relevant analyses were conducted for each ethnic group.
Population characteristics	Population characteristics are summarised in Supplementary Table 1.
Recruitment	The BIDMC cohort<br>The BIDMC cohort is a dataset comprised of routinely collected data from Beth Israel Deaconess Medical Center, Boston, USA. Subject over 16 years old with a valid ECG performed from 2000 to 2023 were included. ECGs were linked to clinical records. In total, 512950 ECGs from 114415 subjects were available for analysis.<br><br>UK Biobank<br>The UK Biobank is longitudinal study of over 500,000 volunteers aged 40-69 at the time of enrolment in 2006-2010 (1). At baseline assessment, participants provided information on health and lifestyle via questionnaire, had physical measures taken (including height, weight, and blood pressure), and donated samples of blood urine and saliva. A subgroup of participants was invited back for subsequent visits for additional investigations, including cardiac magnetic resonance imaging (MRI), brain MRI, and digital ECGs. 42,386 subjects with digital ECGs taken at the instance 2 visit were available for analysis. There is evidence of healthy volunteer selection bias (2). Outcomes were linked to cancer and death registry data, hospital admissions, and primary care records. Detailed phenotyping using the cardiac MRI data has been previously described (3, 4).
Ethics oversight	For the Beth Israel Deaconess Medical Center (BIDMC) cohort ethics review and approval were provided by the Beth Israel Deaconess Medical Center Committee on Clinical Investigations, IRB protocol # 2023P000042.<br><br>The UK Biobank has approval from the North West Multi-Centre Research Ethics Committee as a Research Tissue Bank (application IDs 48666, 47602).

Note that full information on the approval of the study protocol must also be provided in the manuscript.

Field-specific reporting

Please select the one below that is the best fit for your research. If you are not sure, read the appropriate sections before making your selection.

☒ Life sciences ☐ Behavioural & social sciences ☐ Ecological, evolutionary & environmental sciences

For a reference copy of the document with all sections, see nature.com/documents/nr-reporting-summary-flat.pdf

Life sciences study design

All studies must disclose on these points even when the disclosure is negative.

Sample size	All participants meeting inclusion criteria
Data exclusions	Participants excluded if no ECG or Body Mass Index available
Replication	External validation performed in the UK Biobank
Randomization	Observational study - no randomisation
Blinding	Observational study - no randomisation/blinding

Reporting for specific materials, systems and methods

We require information from authors about some types of materials, experimental systems and methods used in many studies. Here, indicate whether each material, system or method listed is relevant to your study. If you are not sure if a list item applies to your research, read the appropriate section before selecting a response.

Materials & experimental systems

n/a	Involved in the study
☒	☐ Antibodies
☒	☐ Eukaryotic cell lines
☒	☐ Palaeontology and archaeology
☒	☐ Animals and other organisms
☐	☒ Clinical data
☒	☐ Dual use research of concern
☒	☐ Plants

Methods

n/a	Involved in the study
☒	☐ ChIP-seq
☒	☐ Flow cytometry
☒	☐ MRI-based neuroimaging

Clinical data

Policy information about clinical studies

All manuscripts should comply with the ICMJE guidelines for publication of clinical research and a completed CONSORT checklist must be included with all submissions.

Clinical trial registration

Not a clinical trial

Study protocol

Not a clinical trial

Data collection

The BIDMC cohort
The BIDMC cohort is a dataset comprised of routinely collected data from Beth Israel Deaconess Medical Center, Boston, USA. Subject over 16 years old with a valid ECG performed from 2000 to 2023 were included. ECGs were linked to clinical records. In total, 512950 ECGs from 114415 subjects were available for analysis.

UK Biobank
The UK Biobank is longitudinal study of over 500,000 volunteers aged 40-69 at the time of enrolment in 2006-2010 (1). At baseline assessment, participants provided information on health and lifestyle via questionnaire, had physical measures taken (including height, weight, and blood pressure), and donated samples of blood urine and saliva. A subgroup of participants was invited back for subsequent visits for additional investigations, including cardiac magnetic resonance imaging (MRI), brain MRI, and digital ECGs. 42,386 subjects with digital ECGs taken at the instance 2 visit were available for analysis. There is evidence of healthy volunteer selection bias (2). Outcomes were linked to cancer and death registry data, hospital admissions, and primary care records. Detailed phenotyping using the cardiac MRI data has been previously described (3, 4).

Outcomes

Cardiometabolic disease, Type 2 Diabetes Mellitus, Hypertension, Lipid disorders

Plants

Seed stocks

Report on the source of all seed stocks or other plant material used. If applicable, state the seed stock centre and catalogue number. If plant specimens were collected from the field, describe the collection location, date and sampling procedures.

Novel plant genotypes

Describe the methods by which all novel plant genotypes were produced. This includes those generated by transgenic approaches, gene editing, chemical/radiation-based mutagenesis and hybridization. For transgenic lines, describe the transformation method, the number of independent lines analyzed and the generation upon which experiments were performed. For gene-edited lines, describe the editor used, the endogenous sequence targeted for editing, the targeting guide RNA sequence (if applicable) and how the editor was applied.

Authentication

Describe any authentication procedures for each seed stock used or novel genotype generated. Describe any experiments used to assess the effect of a mutation and, where applicable, how potential secondary effects (e.g. second site T-DNA insertions, mosiacism, off-target gene editing) were examined.
